# Peripheral T cell profiling reveals downregulated exhaustion marker and increased diversity in lymphedema post-lymphatic venous anastomosis

**DOI:** 10.1016/j.isci.2023.106822

**Published:** 2023-05-06

**Authors:** Hirofumi Imai, Takakazu Kawase, Shuhei Yoshida, Toshiro Mese, Solji Roh, Asuka Fujita, Toshio Uchiki, Ayano Sasaki, Shogo Nagamatsu, Atsushi Takazawa, Tatsuo Ichinohe, Isao Koshima

**Affiliations:** 1 International Center for Lymphedema, Hiroshima University Hospital, Hiroshima 734-8551, Japan; 2 Department of Hematology and Oncology, Research Institute for Radiation Biology and Medicine, Hiroshima University, Hiroshima 734-8553, Japan; 3 International Center for Cell and Gene Therapy, Fujita Health University, Toyoake, Aichi 470-1192, Japan; 4 Department of Plastic and Reconstructive Surgery, Hiroshima University Hospital, Hiroshima 734-8551, Japan; 5 Department of Orthopaedic Surgery, Hiroshima Hiramatsu Hospital, Hiroshima 732-0816, Japan

**Keywords:** Health sciences, Immunology, Components of the immune system

## Abstract

Lymphedema is a progressive condition accompanying cellulitis and angiosarcoma, suggesting its association with immune dysfunction. Lymphatic venous anastomosis (LVA) can provide relief from cellulitis and angiosarcoma. However, the immune status of peripheral T cells during lymphedema and post-LVA remains poorly understood. Using peripheral blood T cells from lymphedema, post-LVA, and healthy controls (HCs), we compared the profile of T cell subsets and T cell receptor (TCR) diversity. PD-1^+^ Tim-3 ^+^ expression was downregulated in post-LVA compared with lymphedema. IFN-γ levels in CD4^+^PD-1^+^ T cells and IL-17A levels in CD4^+^ T cells were downregulated in post-LVA compared with lymphedema. TCR diversity was decreased in lymphedema compared with HCs; such TCR skewing was drastically improved in post-LVA. T cells in lymphedema were associated with exhaustion, inflammation, and diminished diversity, which were relieved post-LVA. The results provide insights into the peripheral T cell population in lymphedema and highlight the immune modulatory importance of LVA.

## Highlights

• We demonstrated the peripheral T cell condition in lymphedema
• The influence of lymphatic venous anastomosis on peripheral T cells was elucidated
• T cells after lymphatic venous anastomosis demonstrated upregulation of diversity
• Our results highlight the importance of LVA apart from its edema-reducing effect

## Graphical abstract

**Figure:**
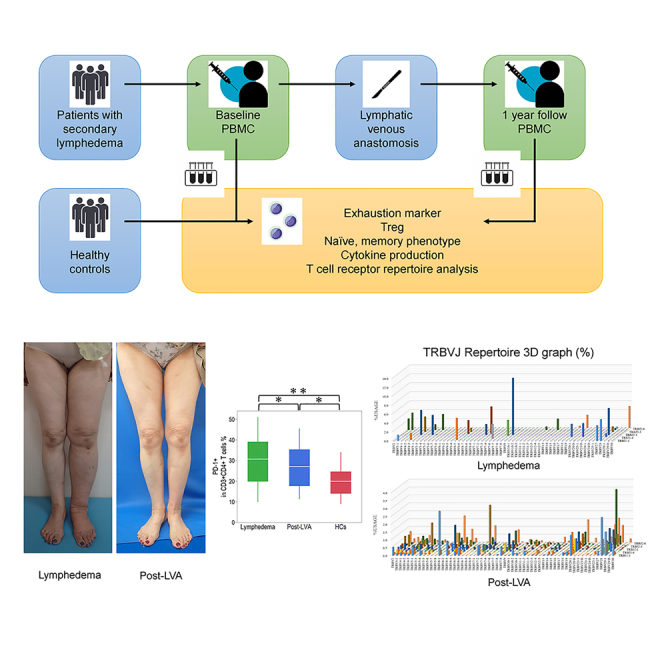

## Introduction

Lymphedema results in the swelling of limbs because of lymph retention following resection, radiotherapy, and lymph node dissection as part of cancer therapy.^1^ It is estimated that 20%–40% of patients who undergo treatment for solid malignancies, such as breast cancer, melanoma, gynecological or urological tumors, or sarcomas, develop lymphedema.^2^ Patients with lymphedema develop progressive fibroadipose deposition in the affected limb and are at an increased risk of developing cellulitis and angiosarcoma, suggesting the coexistence of immune dysfunction.^3^ The incidence of cellulitis in patients with lymphedema is 10%–40%,^4^^,^^5^ and approximately 20% of these patients experience recurrence of cellulitis that occasionally leads to sepsis and mortality.^6^^,^^7^ Angiosarcoma is a rare but fatal tumor that develops 10–15 years after primary cancer therapy, including surgery and radiotherapy.^8^ Even with the treatment available for angiosarcoma, the prognosis remains poor, with the median duration to mortality reported to be as short as 10.5 months.^8^

Lymphatic venous anastomosis (LVA) is a surgical treatment that improves lymphatic drainage by anastomosing the lymphatic vessels to a cutaneous vein under surgical microscopy.^9^ LVA can reduce the circumference of the lymphedematous extremity with low invasiveness, and cellulitis infections are significantly reduced after treatment with LVA.^10^^,^^11^ Notably, Koshima et al. reported the effectiveness of LVA that can treat lymphedema-related angiosarcoma, including lung metastatic lesion.^12^ Increased risk of cellulitis and angiosarcoma has been considered a manifestation of immune dysfunction related to lymphedema, and LVA can influence the immunity of lymphedema.^13^

The analysis of lymphedematous tissue revealed that T cells, particularly Th2 cells, contribute to the development of lymphedema.^14^ Recently, a clinical trial reported on IL-4/IL-13-neutralizing antibodies targeting Th2 cells^15^; treatment with IL-4/IL-13-neutralizing antibodies improved quality of life measurements, skin stiffness, and histological changes in the lymphedematous extremity; however, it appeared to be less effective for the reduction of edema. Hence, lymphedema is intimately associated with the formation of a particular immunological environment, especially that of T cells; however, the immune profiling of peripheral T cell populations in lymphedema has not been investigated comprehensively. To enhance our understanding of the immune status in patients with lymphedema and those who have undergone post-LVA, a more detailed investigation of peripheral T cells is warranted. The principal aim of this study was to elucidate the characteristics of peripheral T cell subpopulations and T cell receptor (TCR) repertoire in patients with lymphedema and investigate the alterations in T cell profiles after treatment with LVA.

## Results

### Patient demographics

**Figure 1:**
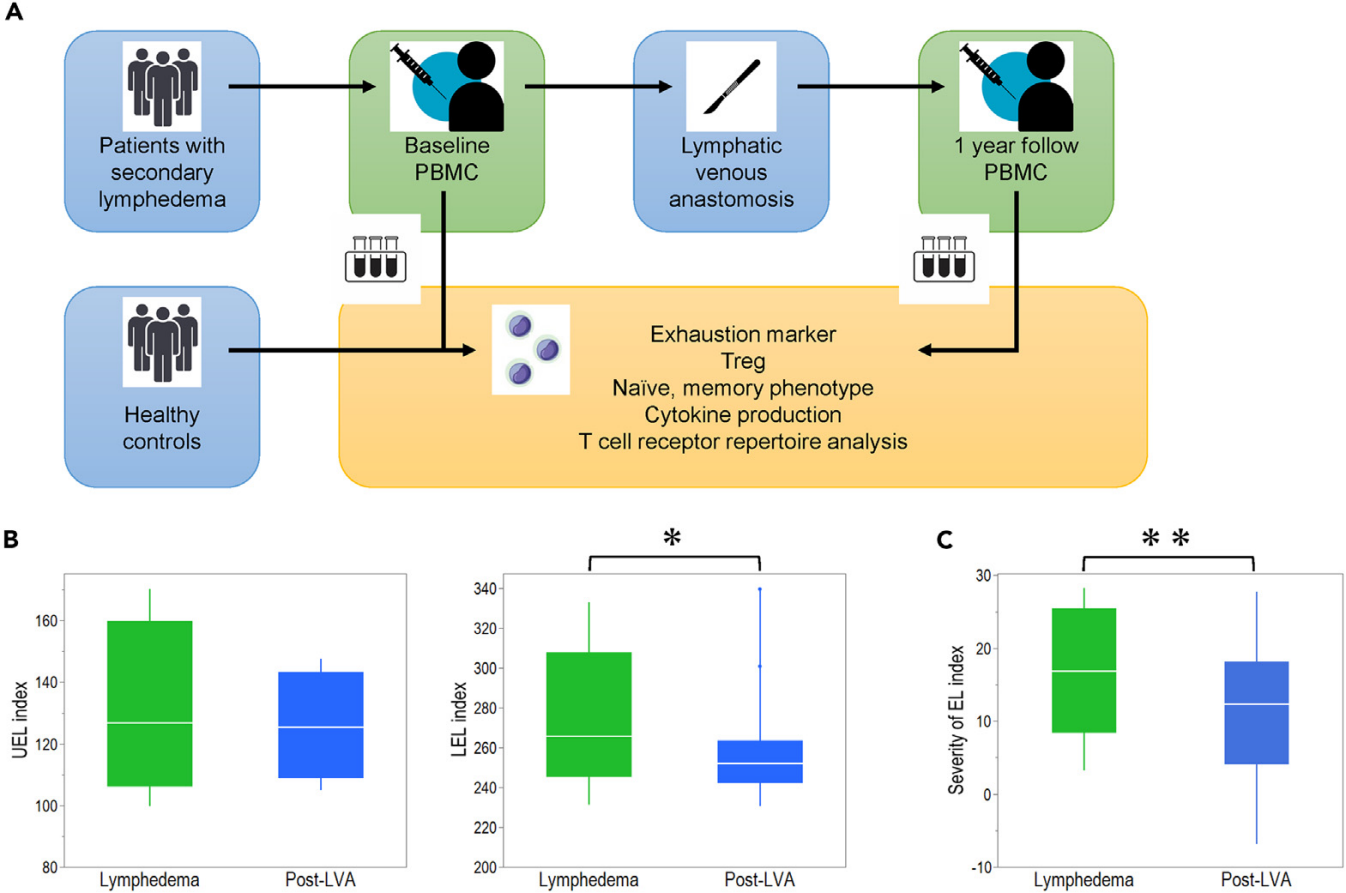
Study design and clinical efficacy (A) Study design of this research. (B) Comparison between lymphedema and post-lymphatic venous anastomosis (LVA) in terms of upper extremity lymphedema (UEL) index (left side) and lower extremity lymphedema (LEL) index (right side). (C) Comparison between lymphedema and post-(LVA) in terms of severity of the EL index. ∗p < 0.05, ∗∗p < 0.01. Non-parametric, independent, and paired continuous variables were compared using Mann–Whitney U test and Wilcoxon’s rank-sum test, respectively. Data are represented as median +/− interquartile range.

Of the 21 enrolled female patients, 17 showed lower extremity lymphedema and 4 showed upper extremity lymphedema (Figure 1A). The median [interquartile range (IQR)] age of the enrolled patients was 54 (45–59.8) years (Table S1), and the median (IQR) BMI was 20.7 (19.2–25.1) kg/m^2^. The most frequently associated illness of the patients was uterine cancer (n = 14, 66.7%), followed by breast cancer (n = 4, 19.0%), ovarian cancer (n = 2, 9.5%), and bladder cancer (n = 1, 4.8%). All patients were classified as International Society of Lymphology stage II to III and dermal backflow stage II to Ⅴ.^16^^,^^17^ The median duration of lymphedema was 2.7 (0.5–7.5) years.

### Clinical efficacy

A median number of 6 (5.5–7) LVAs were performed per patient. The upper extremity lymphedema index in post-LVA decreased compared with that in lymphedema, although it was not significant (126.7 [106.0–159.8] vs. 125.4 [108.9–143.2], p = 0.25) (Figure 1B) (Table S2).^18^ The lower extremity lymphedema index of post-LVA decreased significantly compared with that of lymphedema (265.5 [245.2–307.7] vs. 251.7 [241.9–263.5], p = 0.01).^19^ The severity of extremity lymphedema (EL) index of post-LVA decreased significantly compared with that of lymphedema (16.8 [8.4–25.4] vs. 12.3 [4.1–18.1], p < 0.01) (Figure 1C). Leukocyte count, lymphocyte count, and CD4/CD8 ratio in CD3^+^ cells in peripheral blood were not significantly different among lymphedema, post-LVA, and healthy controls (HCs) (Table S2).

### PD-1, Tim-3, Lag-3, and PD-1^+^Tim-3^+^ expression on peripheral CD4^+^ and CD8^+^ T cells in patients with lymphedema, post-LVA, and HCs

**Figure 2:**
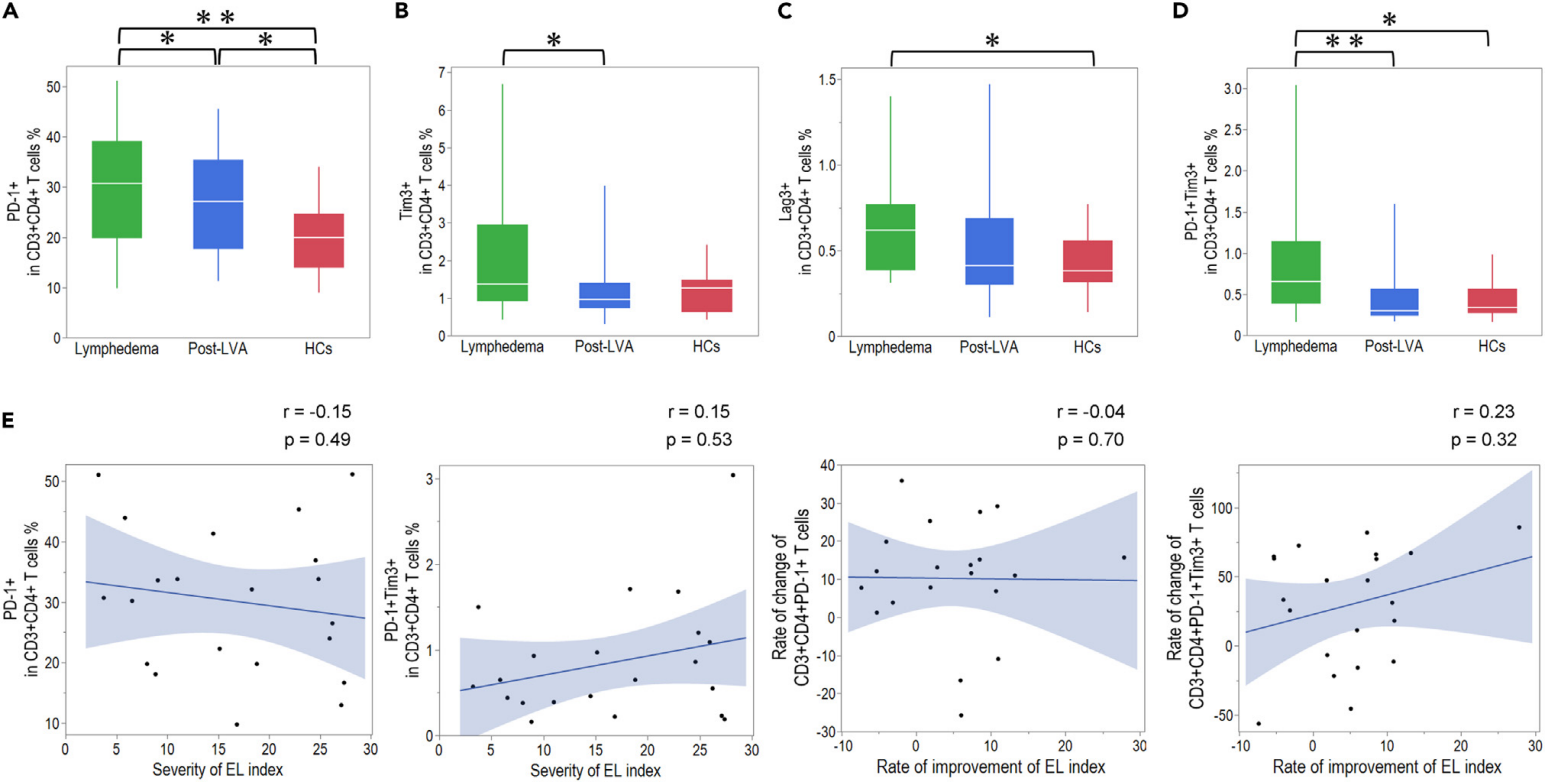
Comparison of the exhaustion marker expression in CD4^+^ T cells among patients with lymphedema, post-lymphatic venous anastomosis (LVA), and healthy controls (HCs) Expression of (A) PD-1, (B) Tim-3, (C) Lag-3, and (D) PD-1^+^Tim3^+^ on CD4^+^ T cells. (E) Correlation between exhaustion marker and severity of the extremity lymphedema (EL) index, rate of change of exhaustion marker, and rate of improvement of the EL index. ∗p < 0.05, ∗∗p < 0.01. Non-parametric, independent, and paired continuous variables were compared using Mann–Whitney U test and Wilcoxon’s rank-sum test, respectively. Data are represented as median +/− interquartile range.

**Figure 3:**
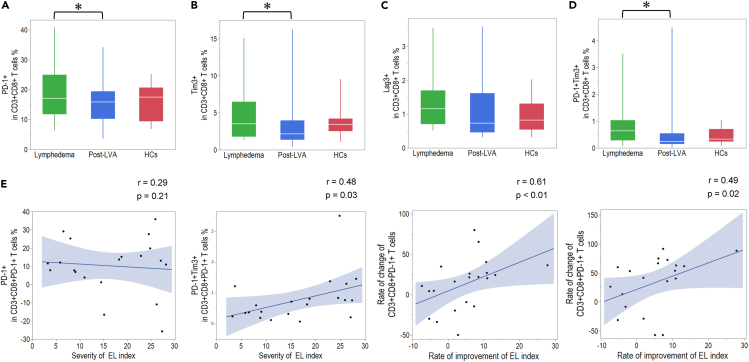
Comparison of exhaustion marker expression in CD8^+^ T cells among patients with lymphedema, post-lymphatic venous anastomosis (LVA), and healthy controls (HCs) Expression of (A) PD-1, (B) Tim-3, (C) Lag-3, and (D) PD-1^+^Tim3^+^ on CD8^+^ T cells. (E) Correlation between exhaustion marker and severity of extremity lymphedema (EL) index, rate of change of exhaustion marker, and rate of improvement of the EL index. ∗p < 0.05. Non-parametric, independent, and paired continuous variables were compared using Mann–Whitney U test and Wilcoxon’s rank-sum test, respectively. Data are represented as median +/− interquartile range.

To understand the role of the exhaustion marker on CD4^+^ T cells in lymphedema, we examined the expression patterns of programmed death-1 (PD-1), T cell immunoglobulin and mucin domain-containing-3 (Tim-3), lymphocyte activation gene-3 (Lag-3), and PD-1^+^Tim-3^+^, which is known as the exhaustion marker, on peripheral CD4^+^ T cells in lymphedema, post-LVA, and HCs. PD-1, Tim-3, and PD-1^+^Tim-3^+^ expression on CD4^+^ T cells showed significant downregulation in post-LVA compared to lymphedema; 30.7 (19.8–39.1)% vs. 27.1 (17.6–35.4)%, p = 0.03; 1.4 (0.9–3.0)% vs. 1.0 (0.7–1.4)%, p < 0.01; and 0.7 (0.4–1.1)% vs. 0.3 (0.2–0.6)%, p < 0.01, respectively (Figures 2A–2D and Table S3). Additionally, PD-1, Lag-3, and PD-1^+^Tim-3^+^ expression on CD4^+^ T cells showed a significant upregulation in patients with lymphedema compared to HCs. Despite the downregulation of PD-1 expression on CD4^+^ T cells in post-LVA compared with lymphedema, PD-1 expression post-LVA remained significantly higher than the expression in HCs. Next, we investigated the correlation between the expression of the exhaustion marker and the clinical severity of lymphedema as well as the correlation between the change in expression of the exhaustion marker and the reduction in edema by LVA. We could not identify any correlation between the expression of the exhaustion marker and the severity of EL index. Furthermore, we could not identify any correlation between the rate of change in the expression of the exhaustion marker and the rate of improvement in the EL index by LVA (Figure 2E). We next examined the expression patterns of PD-1, Tim-3, Lag-3, and PD1^+^Tim-3^+^ on peripheral CD8^+^ T cells in lymphedema, post-LVA, and HCs. PD-1, Tim-3, and PD-1^+^Tim-3^+^ expression on CD8^+^ T cells showed significant downregulation in post-LVA compared to lymphedema; 17.1 (11.7–25)% vs. 15.9 (10.2–19.4)%, p = 0.01; 3.5 (1.8–6.5)% vs. 2.2 (1.3–4.0)%, p < 0.01; and 0.6 (0.3–1.0)% vs. 0.2 (0.1–0.5)%, p = 0.01 (Figures 3A–3D and Table S3), respectively. PD-1^+^Tim-3^+^ expression on CD8^+^ T cells was positively correlated with the severity of the EL index (Figure 3E). The rate of improvement in the EL index was positively correlated with the rate of change in the expression of the exhaustion marker on CD8^+^ T cells.

### Treg population in patients with lymphedema, post-LVA, and HCs

**Figure 4:**
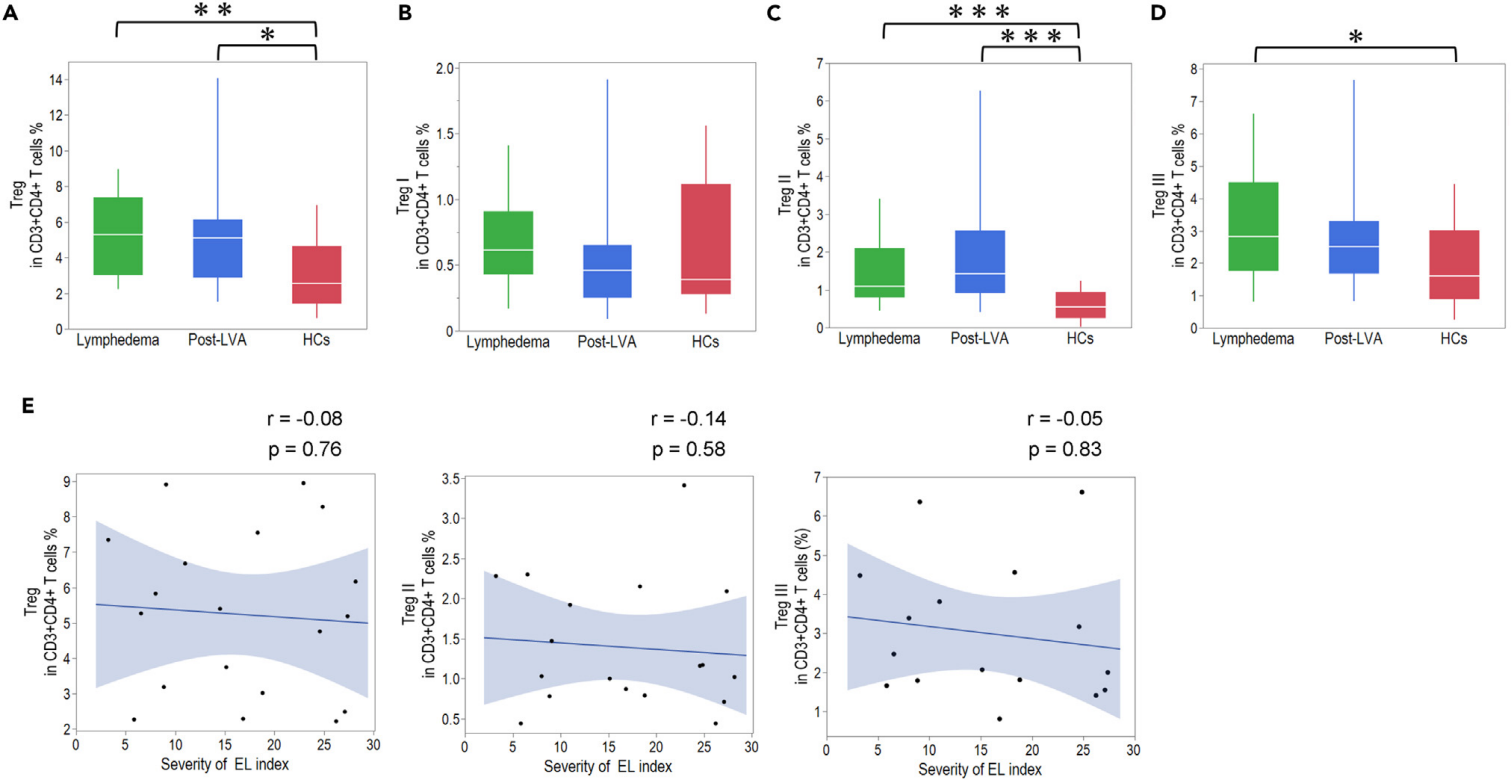
Proportion of Treg subsets in patients with lymphedema, post-lymphatic venous anastomosis (LVA), and healthy controls (HCs) (A) Representative fluorescence-activated cell sorting data of patients with lymphedema and HCs. Comparison of the total proportion of (B) Treg, (C) Treg I, (D) Treg II, and (E) Treg III in CD3^+^CD4^+^ T cells among patients with lymphedema, post-LVA, and HC samples. (F) Correlation between Treg proportion and severity of the extremity lymphedema (EL) index. ∗p < 0.05, ∗∗p < 0.01, ∗∗∗p < 0.001. Non-parametric, independent, and paired continuous variables were compared using Mann–Whitney U test and Wilcoxon’s rank-sum test, respectively. Data are represented as median +/− interquartile range.

We compared Tregs and three distinct subpopulations of Tregs (Treg Ⅰ, Treg Ⅱ, and Treg Ⅲ) between patients with lymphedema, post-LVA, and HCs to understand the relationship between Treg and lymphedema. The frequency of Tregs and the three Treg subpopulations had not changed significantly between lymphedema and post-LVA (Table S4). The total proportion of Tregs (including the proportion of Treg Ⅰ, Treg Ⅱ, and Treg Ⅲ) in CD4^+^ T cells was significantly higher in lymphedema compared to HCs (Figure 4A). Although the proportion of Treg Ⅰ in CD4^+^ T cells was similar between lymphedema and HCs (Figure 4B), it was notable that the proportion of Treg Ⅱ and Treg Ⅲ in CD4^+^ T cells was significantly higher in lymphedema compared to HCs (Figures 4C and 4D). We could not identify any correlation between the Treg populations and the EL severity index (Figure 4E).

### Comparison of naive and memory CD4^+^ T cells between patients with lymphedema, post-LVA, and HCs

**Figure 5:**
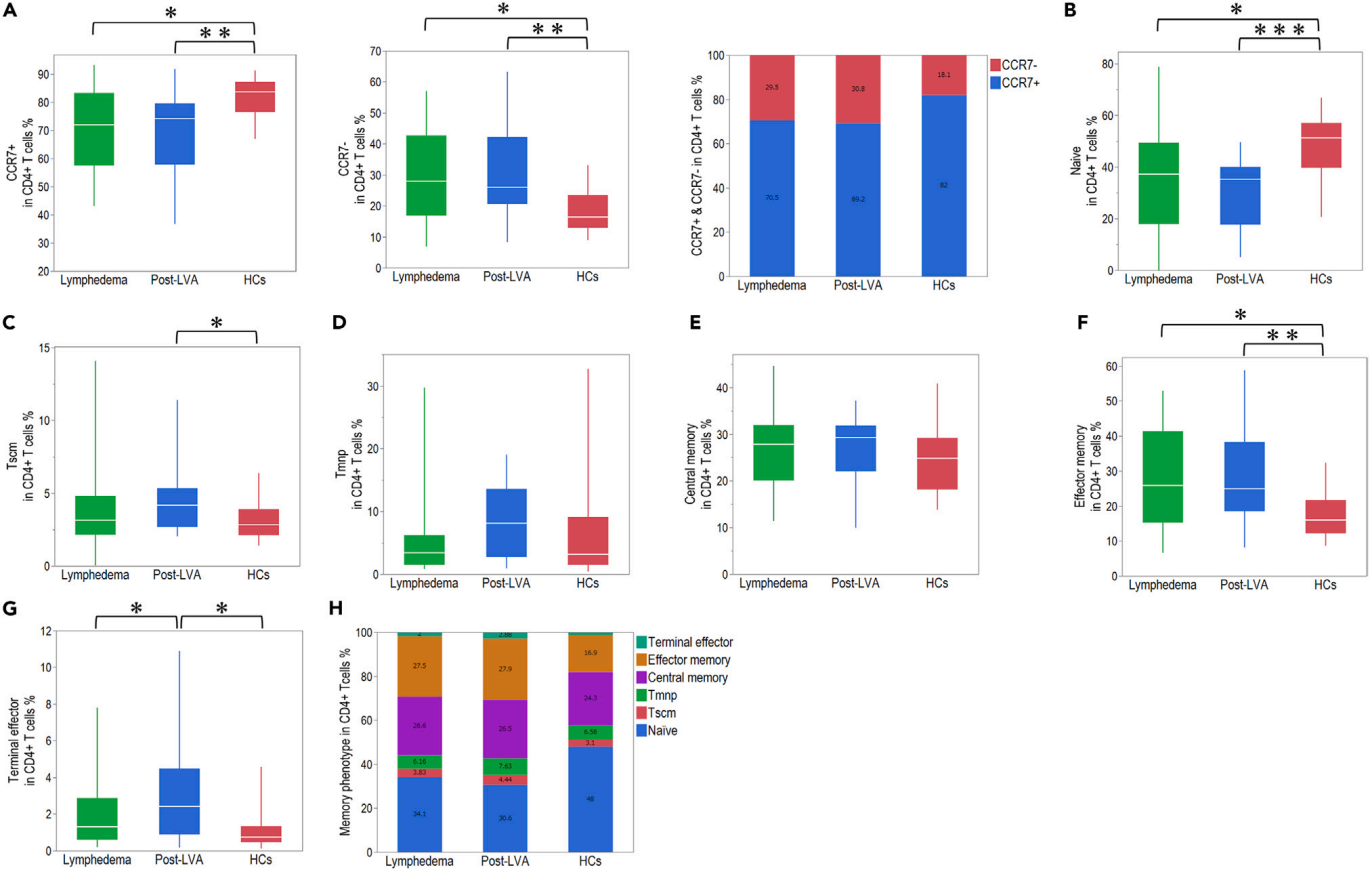
Naive and memory phenotype frequencies on CD4^+^ T cells in patients with lymphedema, post-lymphatic venous anastomosis (LVA), and healthy controls (HCs) (A) Comparison of CCR7^+^ and CCR7^-^ expression in CD4^+^ T cells among patients with lymphedema, post-LVA, and HCs. (B) Naive, (C) stem cell-like memory T cell (Tscm), (D) memory T cells with a naive phenotype (Tmnp), (E) central memory, (F) effector memory, and (G) terminal effector population in CD4^+^ T cells in patients with lymphedema, post-LVA, and HCs. (H) Total demographic of naive and memory phenotype frequencies in CD4^+^ T cells. ∗p < 0.05, ∗∗p < 0.01, ∗∗∗p < 0.001. Non-parametric, independent, and paired continuous variables were compared using Mann–Whitney U test and Wilcoxon’s rank-sum test, respectively. Data are represented as median +/− interquartile range.

**Figure 6:**
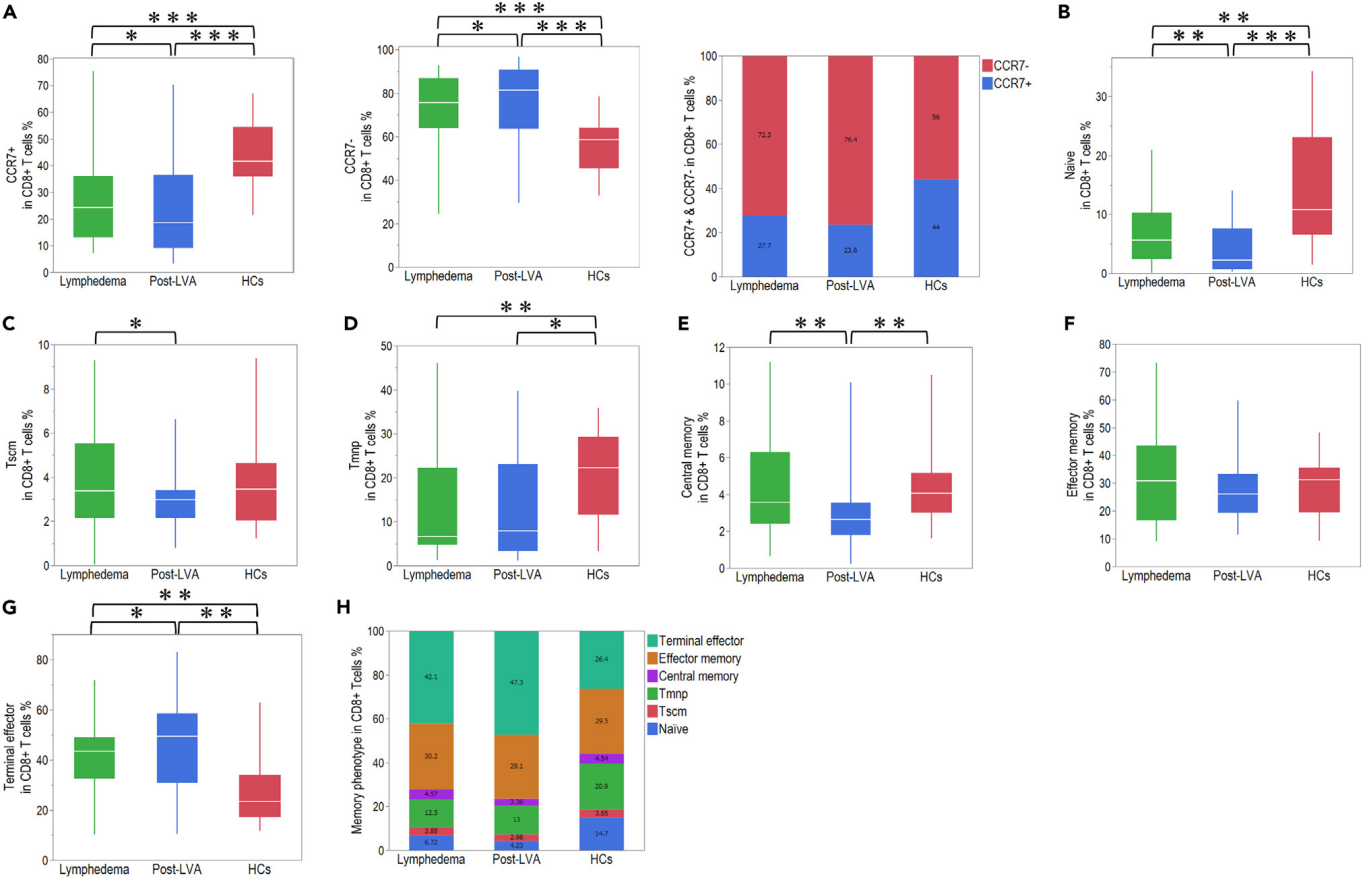
Naive and memory phenotype frequencies on CD8^+^ T cells in patients with lymphedema, post-lymphatic venous anastomosis (LVA), and healthy controls (HCs) (A) Comparison of CCR7^+^ and CCR7^-^ expression in CD8^+^ T cells among patients with lymphedema, post-LVA, and HCs. (B) Naive, (C) stem cell-like memory T cell (Tscm), (D) memory T cells with a naive phenotype (Tmnp), (E) central memory, (F) effector memory, and (G) terminal effector population in CD8^+^ T cells in patients with lymphedema, post-LVA, and HCs. (H) Total demographic of naive and memory phenotype frequencies in CD8^+^ T cells. ∗p < 0.05, ∗∗p < 0.01, ∗∗∗p < 0.001. Non-parametric, independent, and paired continuous variables were compared using Mann–Whitney U test and Wilcoxon’s rank-sum test, respectively. Data are represented as median +/− interquartile range.

To understand the features of the activation state in peripheral CD4^+^ T cells in lymphedema, we compared the proportions of naive and memory CD4^+^ T cells among the total CD4^+^ T cells (Table S5). The number of CCR7^+^CD4^+^ T cells was lower in lymphedema compared with that in HCs, and that the number of CCR7^−^CD4^+^ T cells was higher in lymphedema compared with that in HCs; however, significant changes were not observed between lymphedema and post-LVA (Figure 5A). The proportion of naive, stem cell-like memory T cells (Tscm), memory T cells with naive phenotype (Tmnp), central memory (CM), and effector memory (EM) cells among CD4^+^ T cells was not changed between lymphedema and post-LVA but was accompanied by an increasing proportion of terminal effector (TE) cells in post-LVA (Figures 5B–5H). The proportion of naive T cells was decreased in patients with lymphedema compared with HCs, and the proportion of EM cells was increased in patients with lymphedema compared with HCs. We could not identify any correlation between the populations of naive and memory phenotypes among CD4^+^ T cells and severity of the EL index (Figure S1). We also compared the naive and memory subpopulations among CD8^+^ T cells (Table S5). In contrast to CCR7 expression in CD4^+^ T cells, the number of CCR7^+^CD8^+^ cells was significantly decreased, whereas that of CCR7^−^CD8^+^ cells was significantly increased in post-LVA compared with that in lymphedema (Figure 6A). The population of naive, Tscm, and CM cells was decreased, whereas that of TE cells was increased in post-LVA compared with that in lymphedema (Figures 6B–6H). The population of naive and Tmnp cells was decreased, whereas that of TE cells was increased in patients with lymphedema compared with that in HCs. Similar to that in CD4^+^ T cells, we could not identify any correlation between the population of naive and memory phenotypes in CD8^+^ T cell and severity of the EL index (Figure S2).

### Comparison of cytokine production in CD4^+^ and CD8^+^ T cells among patients with lymphedema, post-LVA, and HCs

**Figure 7:**
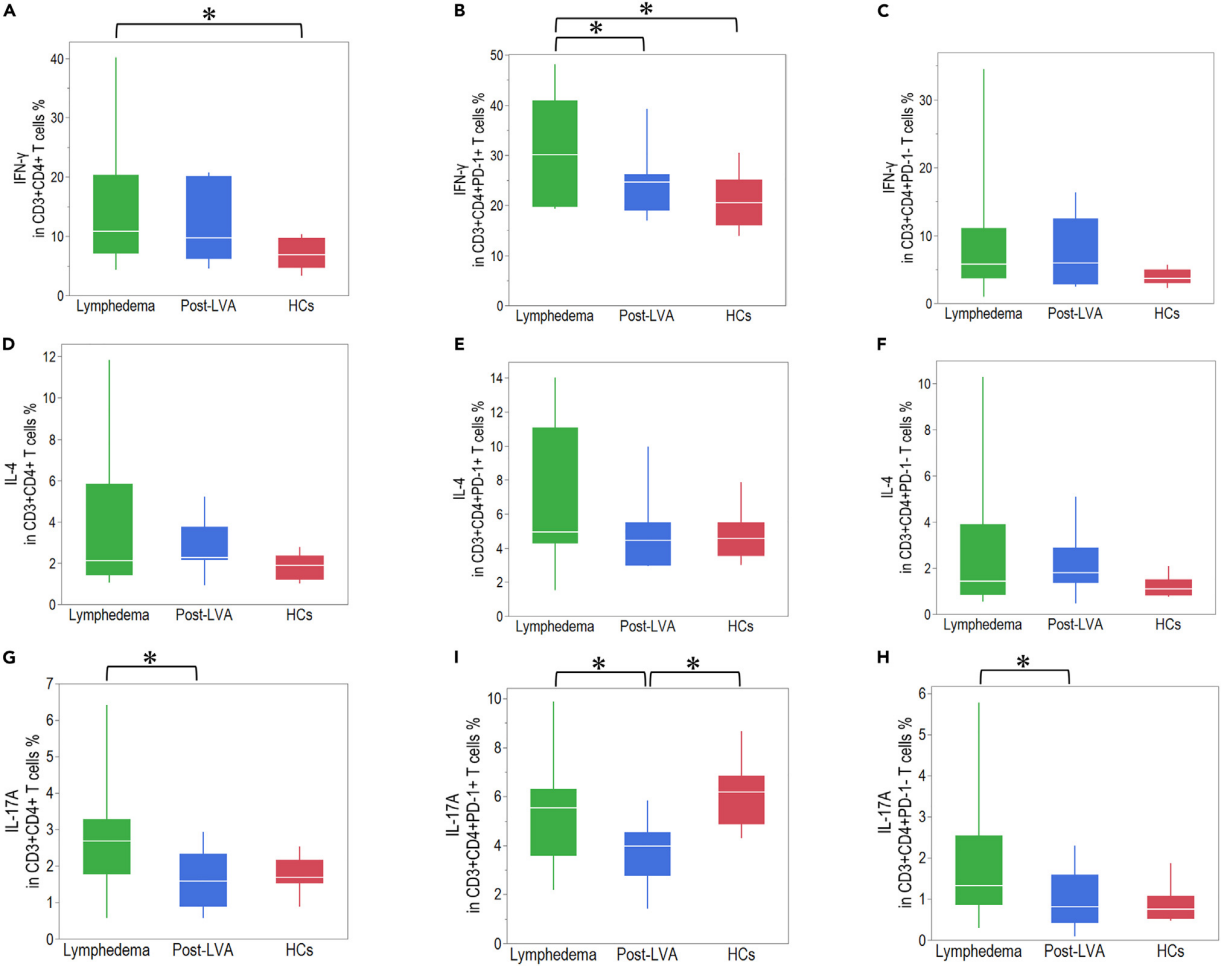
Cytokine production in CD4^+^ T cells in patients with lymphedema, post-lymphatic venous anastomosis (LVA), and healthy controls (HCs) The expression of IFN-γ in (A) CD3^+^CD4^+^ T cells, (B) CD3^+^CD4^+^PD-1^+^ T cells, and (C) CD3^+^CD4^+^PD-1^-^ T cells. The expression of IL-4 in (D) CD3^+^CD4^+^ T cells, (E) CD3^+^CD4^+^PD-1^+^ T cells, and (F) CD3^+^CD4^+^PD-1^-^ T cells. The expression of IL-17A in (G) CD3^+^CD4^+^ T cells, (H) CD3^+^CD4^+^PD-1^+^ T cells, and (I) CD3^+^CD4^+^PD-1^-^ T cells. ∗p < 0.05. Non-parametric, independent, and paired continuous variables were compared using Mann–Whitney U test and Wilcoxon’s rank-sum test, respectively. Data are represented as median +/− interquartile range.

**Figure 8:**
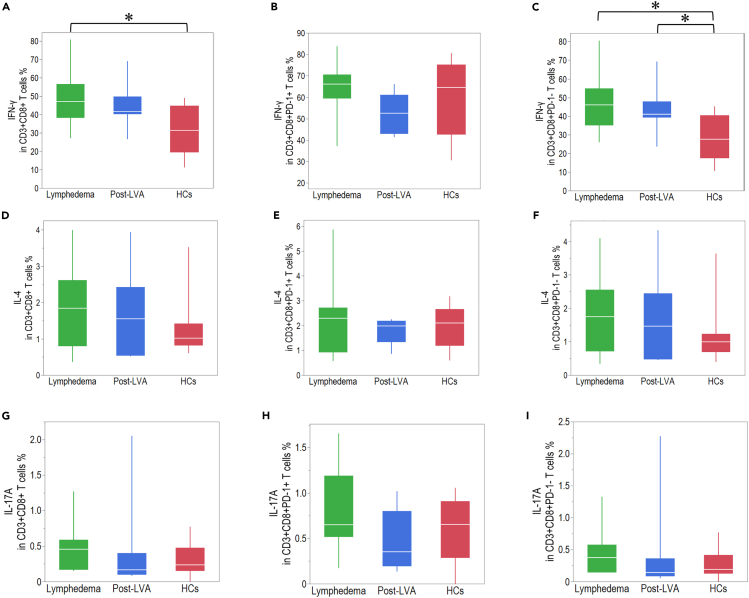
Cytokine production in CD8^+^ T cells in patients with lymphedema, post-lymphatic venous anastomosis (LVA), and healthy controls (HCs) The expression of IFN-γ in (A) CD3^+^CD8^+^ T cells, (B) CD3^+^CD8^+^PD-1^+^ T cells, and (C) CD3^+^CD8^+^PD-1^-^ T cells. The expression of IL-4 in (D) CD3^+^CD8^+^ T cells, (E) CD3^+^CD8^+^PD-1^+^ T cells, and (F) CD3^+^CD8^+^PD-1^-^ T cells. The expression of IL-17A in (G) CD3^+^CD8^+^ T cells, (H), CD3^+^CD8^+^PD-1^+^ T cells, and (I) CD3^+^CD8^+^PD-1^-^ T cells. ∗p < 0.05. Non-parametric, independent, and paired continuous variables were compared using Mann–Whitney U test and Wilcoxon’s rank-sum test, respectively. Data are represented as median +/− interquartile range.

Inflammatory cytokines are strongly involved with the progression of lymphedema.^20^ To understand the correlation between cytokine production by T cells and lymphedema, we compared the expression of IFN-γ, IL-4, and IL-17A on CD4^+^ and CD8^+^ T cells in lymphedema, post-LVA, and HCs (Table S6). IFN-γ expression in CD4^+^PD-1^+^ T cells was downregulated in post-LVA compared with that in lymphedema (30.1 [19.7–40.9] vs. 24.7 [19.0–26.1], p = 0.04). In contrast, IFN-γ expression in CD4^+^PD-1^-^ cells was similar between lymphedema and post-LVA (Figures 7A–7C). No significant difference was noted in IL-4 production in CD4^+^ T cells between lymphedema and post-LVA (Figures 7D–7F). IL-17A production in CD4^+^, CD4^+^PD-1^+^, and CD4^+^PD-1^-^ cells was downregulated in post-LVA compared to that in lymphedema (2.7 [1.8–3.3] vs. 1.6 [0.9–2.3], p = 0.01; 5.5 [3.6–6.3] vs. 4.0 [2.7–4.5], p = 0.04; and 1.3 [0.8–2.5] vs. 0.8 [0.4–1.6], p = 0.01, respectively) (Figures 7G–7I). Cytokine production in CD8^+^ T cells was not significantly different between lymphedema and post-LVA (Figures 8A–8I). In comparison to that in HCs, the IFN-γ production in CD4^+^, CD4^+^PD-1^+^, CD8^+^, and CD8^+^PD-1^-^ T cells was significantly upregulated in patients with lymphedema (Figures 7A, 7B and 8A and 8C).

### Comparison of diversity of TCR β repertoire among patients with lymphedema, post-LVA, and HCs

**Figure 9:**
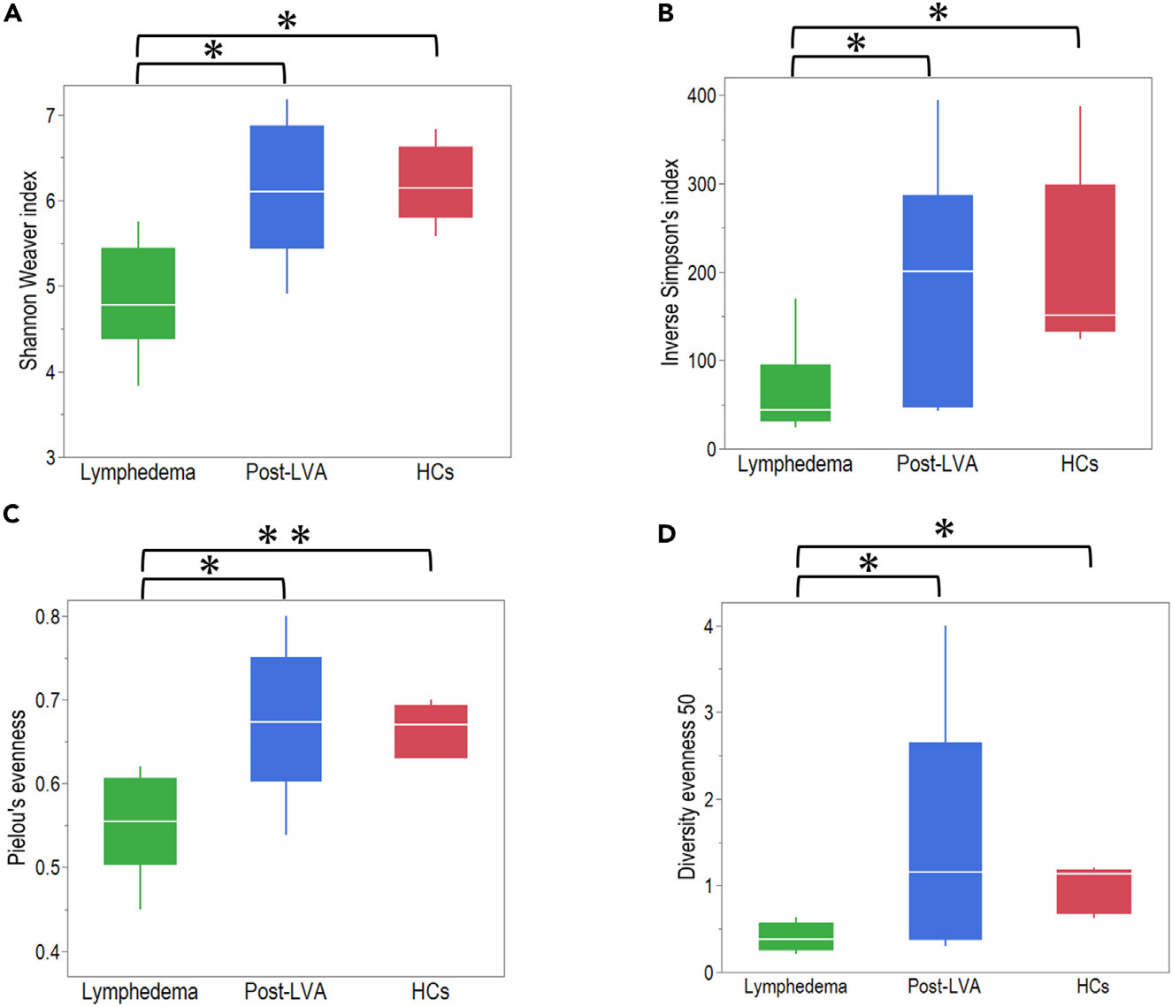
Comparison of diversity of TCR β repertoire among patients with lymphedema, post-lymphatic venous anastomosis (LVA), and healthy controls (HCs) (A) Shannon–Weaver index, (B) inverse Simpson’s index, (C) Pielou’s evenness, and (D) diversity evenness 50 were demonstrated. ∗p < 0.05. Non-parametric, independent, and paired continuous variables were compared using Mann–Whitney U test and Wilcoxon’s rank-sum test, respectively. Data are represented as median +/− interquartile range.

To understand the diversity of peripheral T cells in lymphedema, we analyzed the TCR β repertoire using peripheral blood mononuclear cells in patients with lymphedema, post-LVA, and HCs. No significant difference was noted in sequence reads among patients with lymphedema, post-LVA, and HCs, except that unique reads were significantly lower in patients with lymphedema and those with post-LVA compared to HCs (Table S7). The Shannon–Weaver index, inverse Simpson’s index, Pielou’s evenness, and diversity evenness 50 showed significantly increasing diversity in post-LVA compared with patients with lymphedema (4.7 [4.3–5.4] vs. 6.1 [5.4–6.9], p = 0.02; 44.1 [30.6–95.1] vs. 200.5 [47.2–287], p = 0.02; 0.56 [0.50–0.61] vs. 0.67 [0.60–0.75], p = 0.02; 0.4 [0.2–0.6] vs. 1.2 [0.4–2.7], p = 0.02, respectively) (Figures 9A–9D). Furthermore, these indices indicated a significantly lower diversity of T cells in patients with lymphedema compared with that in HCs. The diminished variety of TRBV and J combination in each patient with lymphedema compared with post-LVA and HCs is shown in Figure S3.

### Expression profile of the TCR β clones in patients with lymphedema, post-LVA, and HCs

**Figure 10:**
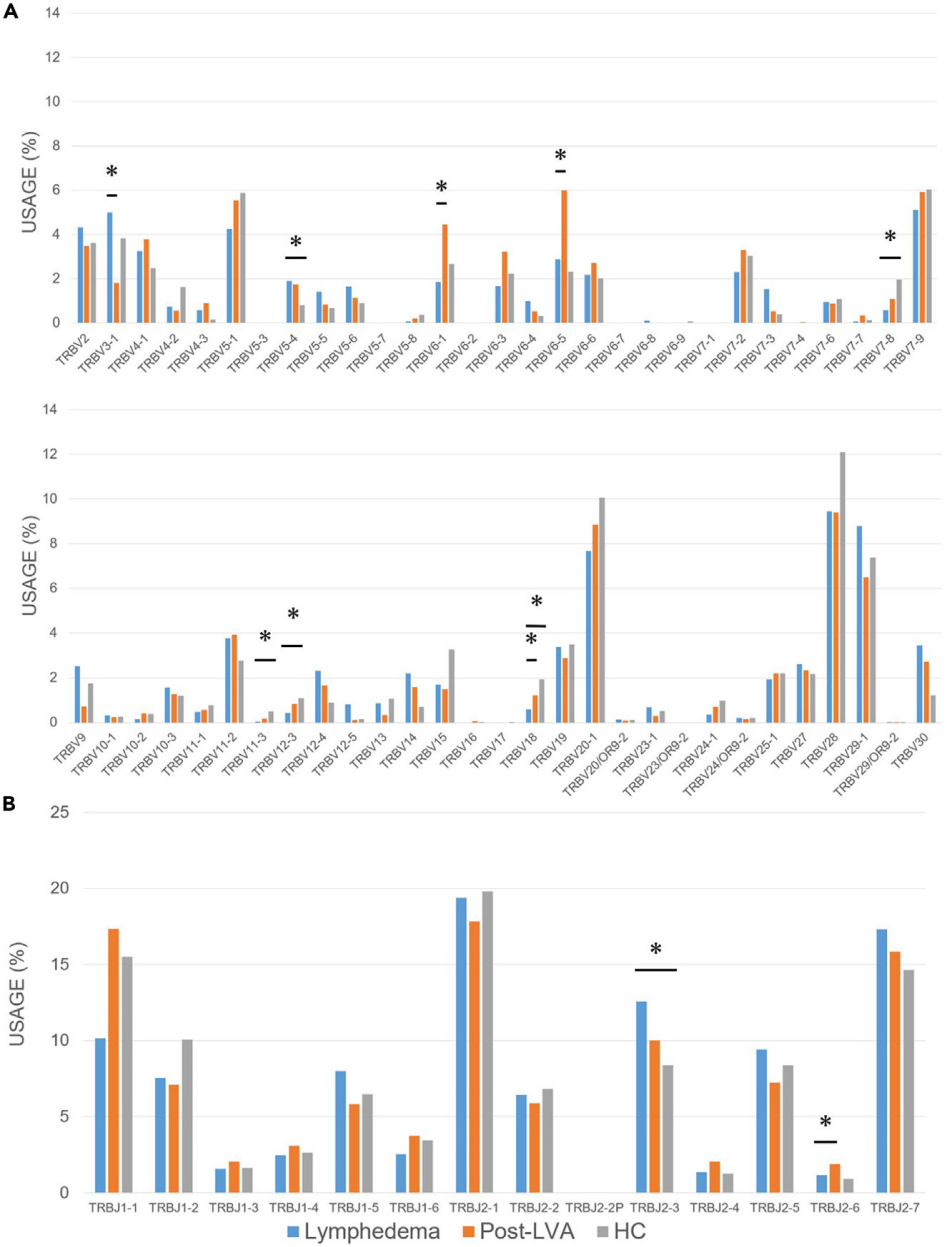
Expression profile of the TCR β clones in patients with lymphedema, post-lymphatic venous anastomosis (LVA), and healthy controls (HCs) Comparison of the usage of (A) TRBV gene and (B) TRBJ gene were demonstrated. ∗p < 0.05. Non-parametric, independent, and paired continuous variables were compared using Mann–Whitney U test and Wilcoxon’s rank-sum test, respectively.

To examine the features of the TCR *β* clonotypes that may characterize the immune environment of lymphedema, we compared the frequency of TRBV and TRBJ in patients with lymphedema, post-LVA, and HCs (Figures 10A and 10B). The frequency of TRBV3-1 was reduced, whereas that of TRBV6-1, 6-5, and 18, and TRBJ2-6 was increased in patients with post-LVA compared with that in lymphedema. With regard to combinations of TRBV and TRBJ, three combinations (TRBV5-1/J1-1, TRBV5-1/J2-7, and TRBV29-1/J1-2) reduced TRB in lymphedema compared with post-LVA. In addition, the three combinations reduced TRB clonotypes in patients with lymphedema compared with that in HCs. The TRBV5-1/J1-1 and TRBV5-1/J2-7 combinations were reduced in patients with lymphedema compared with that in HCs and increased in post-LVA compared with that in lymphedema. We could not identify specific increased clones in lymphedema. The increased and reduced TCR clones between lymphedema and post-LVA are noted in Tables S8 and S9.

## Discussion

In the present study, we analyzed fresh peripheral blood samples and characterized the phenotypes and functional profiles of T cells with comprehensive analysis of the TCR repertoire in patients with lymphedema through post-LVA as a consequence of surgical cancer treatment. In the past, several studies have indicated that local T cells deposited in the tissue are related to the progression of lymphedema.^21^^,^^22^^,^^23^^,^^24^ In particular, Th2-biased inflammatory responses in lymphedema tissue promote tissue fibrosis via increased collagen deposition and aggravate inflammation.^14^ In contrast, increased Treg deposition in lymphedema tissue ameliorates lymphedema development and inflammation.^25^^,^^26^ In a human analysis of the influence of LVA on lymphedema, LVA reduced the proportion of CD4^+^ T cells in lymphedematous tissue, which may be directed back into circulation in the peripheral blood.^27^ Thus, the association between tissue-deposited local T cells and lymphedema inflammation and progression is strongly suggested. However, to the best of our knowledge, no study has reported on the relationship between systemic T cells and lymphedema. Peripheral T cells in lymphedema are influenced by the history of cancer treatment, which makes the study of peripheral T cells in lymphedema more difficult. However, the comparison between lymphedema and post-LVA made it possible to analyze whether the difference in expression in T cells is due to the influence of lymphedema.

The PD-1 regulatory pathway plays indispensable roles in downregulating the immune response and in promoting tolerance to self-antigens by suppressing T cell activation through B7-CD28 co-stimulatory molecules that deliver critical inhibitory signals.^28^ Tim-3, a member of the T cell Ig and mucin domain-containing molecule superfamily, is a key regulatory molecule for the Th1 response.^29^ PD1^+^ Tim-3^+^ co-expressing T cells exhibit reduced proliferation and impaired cytokine production, which is called terminal exhaustion.^30^ Lag-3 exhibits high affinity to major histocompatibility complex class II and regulates the proliferation, activation, and function of T cells.^31^ In the present study, the expression of PD-1, Tim-3, and PD-1^+^Tim-3^+^ on CD4^+^ and CD8^+^ T cells in lymphedema was significantly downregulated in post-LVA, which still showed upregulated expression, compared with that in HCs. Two theories can be considered to explain the upregulation of the exhaustion marker in patients with lymphedema: (1) Patients with lymphedema have increased oxidative stress, which may cause T cell exhaustion.^30^^,^^31^^,^^32^ Yang et al. demonstrated that LVA decreases oxidative stress in the serum of lymphedema.^33^ The upregulation of exhaustion markers in patients with lymphedema is downregulated post-LVA; the phenomenon may reflect the downregulation of oxidative stress in lymphedema through LVA; (2) Previous studies on the immunopathology of lymphedema have demonstrated that CD4^+^ T cells play a role in aggravating tissue fibrosis and lymphatic dysfunction. Upregulated exhaustion markers on CD4^+^ T cell populations in lymphedema might reflect not only chronic consumption of effector CD4^+^ T cells but also the counterbalancing enhancement of suppressed function to inhibit the progress of tissue inflammation and fibrosis. The downregulation of the exhaustion marker on CD4^+^ T cells had no correlation with improvement in the EL index through LVA, whereas the downregulation of the exhaustion marker on CD8^+^ T cells showed a correlation with the improvement of the EL index.

Treg cells compete for the T cell growth factor IL-2 via the expression of high-affinity IL-2 receptor complexes and exert direct suppressive activity by secreting immunosuppressive cytokines such as TGF-β and IL-10.^34^ Treg Ⅰ cells proliferate themselves upon T cell receptor stimulation and convert to Treg Ⅱ cells.^35^ The Treg Ⅱ subset is functionally important with regard to its potent suppressive function, which is related to its high expression of CTLA-4 and CD25 and its higher sensitivity to IL-2 than that of other Treg subpopulations. Treg Ⅲ secretes a high amount of effector cytokines (IL-2, IL-17, and IFN-γ) without suppressive activity. Treg Ⅲ cells may be a heterogeneous subset between Treg cells and effector T cells.^35^ The proportion of peripheral Treg cells is increased in patients with cancer even after tumor resection compared with HCs.^36^ In the present study, Treg, in particular Treg Ⅱ, was upregulated in patients with lymphedema; however, the upregulation was not significantly changed post-LVA. In this study, all patients with lymphedema had undergone cancer treatment. The upregulation of Treg in patients with lymphedema may be influenced by the history of cancer treatment.

Recent evidence suggests that by guiding cells to and within lymphoid organs, CCR7 essentially contributes to both immunity and tolerance.^37^ Furthermore, CCR7^+^ expression is downregulated with aging, chronic inflammation, and experience of cancer treatment.^38^^,^^39^^,^^40^ In the present study, CCR7^+^ expression in CD8^+^ T cells was downregulated in post-LVA compared with that in lymphedema. In contrast, CCR7^+^ expression was not significantly changed in CD4^+^ T cells. The downregulation of CCD7^+^ expression in CD8^+^ T cells may be influenced by aging. In the present study, the number of naive T cells was significantly lower in patients with lymphedema compared to age-matched HCs. However, the depletion of naive T cells was not correlated with lymphedema severity. Hence, the depletion of naive T cells in patients with lymphedema may be influenced by the fact that patients have undergone cancer treatment. Tmnp in CD8^+^ T cells, which express increased levels of CD49d and CXCR3, produce cytokines but maintain a naive phenotype.^41^ The frequency of Tmnp increases with age and after severe acute infection; however, the frequency of Tmnp in this study was reduced in patients with lymphedema and post-LVA compared with HCs.^41^ Further sequencing of this population is warranted to detect the cause of this reduction in the frequency of Tmnp in patients with lymphedema.

The inflammatory cytokines IFN-γ, IL-4, and IL-17A have been reported to negatively control the formation of lymphatic vessels involved in the progression of lymphedema.^20^^,^^42^^,^^43^ Furthermore, Th1, Th2, and Th17 cells are reported to be correlated with the progression of fibrosis and fibrotic diseases.^44^ Th1 cells are considered to play both pro-fibrotic and anti-fibrotic roles, while Th2 and Th17 cells are considered to play dominantly pro-fibrotic roles.^44^ Elevated Th1 cytokine expression inhibits Th2-dominated immune response; however, Th1 cell infiltration leads to cardiac fibrosis.^44^ In patients with lymphedema, Th2 cells in lymphedematous skin are considered to play an important role in the progression of lymphedema.^14^ The analysis of plasma without stimulation could not reveal a significant difference in IFN-γ, IL-4, and IL-17A, whereas some other cytokines (such as MIP-1b and IL-6) were found to be predictive markers of lymphedema when comparing breast cancer-related lymphedema and breast cancer without lymphedema.^45^^,^^46^ We examined the inflammatory cytokine production in CD4^+^ T cells between lymphedema and post-LVA and found that IFN-γ in CD4^+^PD-1^+^ T cells and IL-17A in CD4^+^ T cells were downregulated post-LVA compared with that in lymphedema, while IL-4 in CD4^+^ T cells was not significantly different. It is plausible that IFN-γ has both pro-fibrotic roles for progression of lymphedema and anti-fibrotic roles for counter balancing the Th2-dominant milieu in lymphedematous skin. Hence, IFN-γ is the most affected cytokine by lymphedema. Furthermore, inflammatory cytokine production from CD4^+^ T cells indicated that LVA can ameliorate chronic inflammation in lymphedema.

T cell diversity is required for protective immune responses.^47^ Elderly patients with common variable immunodeficiency or autoimmune diseases show decreased T cell diversity in peripheral blood.^47^^,^^48^^,^^49^ Diminished T cell diversity is thought to result in impaired antipathogen responses.^50^ In the present study, compared with HCs, patients with lymphedema were associated with decreased TCR repertoire diversity, and this TCR skewing was drastically improved post-LVA. These results suggest that LVA can restore the TCR repertoire diversity in lymphedema and thus promote antipathogen responses. This phenomenon may also explain the mechanisms via which LVA reduces the frequency of cellulitis and provides relief from angiosarcoma in lymphedema.^10^^,^^11^^,^^12^ Among the TCR b chains in the repertoire, we observed less frequent usage of TRBV5-1/J1-1 and TRBV5-1/J2-7 in patients with lymphedema, and its restoration to a pattern similar to that in HCs after LVA. These findings of diminished TCR repertoire diversity and decreased specific TCR chains might enhance our understanding of the immunodeficiency in patients with lymphedema.

To the best of our knowledge, this is the first study to demonstrate the peripheral T cell condition in lymphedema and to elucidate the influence of LVA on peripheral T cells. The upregulation of the exhaustion marker, IFN-γ, and IL-17A and downregulation of the TCR repertoire diversity in patients with lymphedema compared with post-LVA and HCs can be associated with immune dysfunction and progression of lymphedema. T cells in lymphedema tend to lead to exhaustion and inflammation with diminishing diversity, which may be associated with immune dysfunction in lymphedema. Notably, LVA can release the exhaustion and inflammation status with upregulation of the diversity of T cells; therefore, this study highlights an advantage of LVA apart from its edema-reducing effect.

### Limitations of the study

This study has some limitations. First, the relatively small number of patients consisting entirely of females is a cause of bias in this study. Second, 16 patients (76.2%) in this study had undergone chemotherapy. Waidhauser et al. reported that chemotherapy did not alter the population of T cells, while Krantz et al. reported that neoadjuvant chemotherapy reduced the expression of CD8^+^ exhaustion marker and the frequency of Treg.^51^^,^^52^ The influence of chemotherapy on circulating T cells is not well known; hence, a future lymphedema study completed the background of chemotherapy is warranted. Third, contracting COVID-19 or receiving a vaccination for COVID-19 has a possible influence on TCR diversity for the timing of this study. We excluded participants with a history of COVID-19 from this study; however, we could not eliminate the possible effect of anti-COVID immune responses during the pandemic.^53^ Fourth, we did not analyze similar T cell profiling data of age-matched patients with cancer but without lymphedema. However, we investigated T cell profiling of patients with lymphedema and after treatment of lymphedema; hence, it is plausible that the comparison of patients with lymphedema and post-LVA indicates the T cell profiling influenced by lymphedema. Yang et al. reported that lymphedema leads to dysregulated gene expression in circulating monocytes, which was restored post-LVA.^54^ Lymphedema is not only a localized disease but also accompanied by systemic disease and LVA can restore healthy condition. A large-scale comprehensive analysis of systemic and local effects of lymphedema with regard to immunology is warranted for understanding the pathophysiology of lymphedema and developing new treatments.

## STAR★Methods

### Key resources table

**Table:** 

REAGENT or RESOURCE	SOURCE	IDENTIFIER
**Antibodies**		

FITC anti-human CD3 (clone: UCHT1)	Biolegend	Cat#300406; RRID: AB_314060
BV510 anti-human CD4 (clone: RPA-T4)	Biolegend	Cat#300546; RRID: AB_2563314
APC/Cy7 anti-human CD4 (clone: RPA-T4)	Biolegend	Cat#300518; RRID: AB_314086
PerCP anti-human CD8 (clone: SK1)	Biolegend	Cat#344708; RRID: AB_1967149
PE/Cy7 anti-human CD8 (clone: SK1)	Biolegend	Cat#344712; RRID: AB_2044008
APC anti-human PD-1 (clone: EH12.2H7)	Biolegend	Cat#329908; RRID: AB_940475
BV421 anti-human Tim3 (clone: F38-2E2)	Biolegend	Cat#345008; RRID: AB_11218598
PE anti-human Lag3 (clone: 11C3C65)	Biolegend	Cat#369306; RRID: AB_2629592
BV421 anti-human CD45RA (clone: HI100)	Biolegend	Cat#304130; RRID: AB_10965547
PE anti-human FOXP3 (clone: 206D)	Biolegend	Cat#320108; RRID: AB_492986
BV510 anti-human CCR7 (clone: G043H7)	Biolegend	Cat#353232; RRID: AB_2563866
FITC anti-human CD45RO (clone: UCHL1)	Biolegend	Cat#304242; RRID: AB_2564159
APC anti-human CD95 (clone: DX2)	Biolegend	Cat#305612; RRID: AB_314550
BV421 anti-human CXCR3 (clone: G025H7)	Biolegend	Cat#353716; RRID: AB_2561448
PE anti-human CD49d (clone: 9F10)	eBioscience	Cat#12049942; RRID: AB_10717245
PE/Cy7 anti-human IFN-γ (clone: 4S.B3)	Biolegend	Cat#502527; RRID: AB_1626154
PE anti-human IL-4 (clone: 8D4-8)	eBioscience	Cat#12704941; RRID: AB_1548823
BV421 anti-human IL-17A (clone: BL168)	Biolegend	Cat#512321; RRID: AB_10899566
FITC Mouse IgG2a κ isotype Ctrl (clone: MOPC-21)	Biolegend	Cat#400210; RRID: AB_326458
BV510 Mouse IgG2b κ isotype Ctrl (clone: MOPC-21)	Biolegend	Cat#400172; RRID: AB_2714004
PerCP Mouse IgG1 κ isotype Ctrl (clone: MOPC-21)	Biolegend	Cat#400148; RRID: AB_893680
APC Mouse IgG1 κ isotype Ctrl (clone: MOPC-21)	Biolegend	Cat#400122; RRID: AB_326443
BV421 Mouse IgG1 κ isotype Ctrl (clone: MOPC-21)	Biolegend	Cat#400158; RRID: AB_11150232
PE Mouse IgG1 κ isotype Ctrl (clone: MOPC-21)	Biolegend	Cat#400114; RRID: AB_2847829
PE/Cy7 Mouse IgG1 κ isotype Ctrl (clone: MOPC-21)	Biolegend	Cat#400125; RRID: AB_2861433
PE Rat IgG1 κ isotype Ctrl (clone: MOPC-21)	Biolegend	Cat#400407; RRID: AB_326513
BV421 Mouse IgG2b κ isotype Ctrl (clone: MOPC-21)	Biolegend	Cat#400342; RRID: AB_2935627

**Bacterial and virus strains**		

*E. coli* DNA polymerase I	Invitrogen	Cat#18010-025
*E. coli* DNA Ligase	Invitrogen	Cat#18052-019

**Biological samples**		

Whole blood from patients with lymphedema	Hiroshima University Hospital, Hiroshima Hiramatsu Hospital	N/A
Whole blood from healthy human	Hiroshima University Hospital	N/A

**Chemicals, peptides, and recombinant proteins**		

7-Aminoactinomycin D	Biolegend	Cat#420403
Zombie-NIR	Biolegend	Cat#423105
RNase H	Invitrogen	Cat#18021-071
T4 DNA polymerase	Invitrogen	Cat#18005-025

**Critical commercial assays**		

True-Nuclear Transcription Factor Buffer Set	Bbiolegend	Cat#424401
Cell Activation Cocktail (with Brefeldin A)	Biolegend	Cat#423303
Cyto-Fast Fix/Perm Buffer Set	Biolegend	Cat#426803
RNeasy Plus Universal Mini Kit	Qiagen	Cat#73404
Superscript III reverse transcriptase	Invitrogen	Cat#18080-085
NotI site	Takara Bio	Cat#1166B
NotI restriction enzyme	Takara Bio	Cat#1246B
KAPA 578 HiFi DNA Polymerase	Kapa Biosystems	Cat#KK2602
Nextera XT index kit v2 setA or setD	Illumina	Cat#FC-131-2001
Qubit 3.0 Fluorometer	Thermo Fisher Scientific	Cat#Q33216
Lymphoprep	STEMCELL Technologies	Cat#07851

**Deposited data**		

T cell receptor repertoire sequences	This study	Zenodo (https://doi.org/10.5281/zenodo.7854980)

**Oligonucleotides**		

P10EA adaptor (GGGAATTCGG)	Invitrogen	N/A
P20EA primers (TAATACGACTCCGAATTCCC)	Invitrogen	N/A
P22EA-ST1-R primers (GTCTCGTGGGCTCGGAGATG TGTATAAGAGACAGCTAATACGACTCCGAATTCCC)	Invitrogen	N/A
BSL-18E primer containing polyT18 (AAAGCG GCCGCATGCTTTTTTTTTTTTTTTTTTVN)	Invitrogen	N/A
TCR β 1st PCR CB1(2) (GAACTGGACTTGACA GCGGAACT)	This study	N/A
TCR β 2nd PCR CB2 (AGGCAGTATCTGGAGT CATTGAG)	This study	N/A
TCR β Tag PCR CB-ST1-R (TCGTCGGCAGCGTCAGAT GTGTATAAGAGACAGGCTCAAACACAGCGACCTC)	This study	N/A

**Software and algorithms**		

BD FACSDiva	BD Biosciences	N/A
FlowJo (v10.8.1)	FlowJo, LLC, BD Biosciences	https://www.flowjo.com/
Repertoire analysis software Repertoire Genesis	Repertoire Genesis Inc.	N/A
JMP Pro 16	JMP Statistical Discovery LLC.	https://www.jmp.com/en_us/home.html
Repertoire Genesis	Kitaura, et al.^55^^,^^56^	N/A

**Other**		

FACS Canto II	BD Biosciences	N/A

### Resource availability

#### Lead contact

Further information and requests for resources and reagents should be directed to and will be fulfilled by the lead contact, Hirofumi Imai (imai_h61@yahoo.co.jp).

#### Materials availability

This study did not generate new unique reagents.

### Experimental model and subject details

#### Patients and healthy controls

This prospective study included patients with a diagnosis of lymphedema and age- and sex-matched HCs without a history of cancer. All participants were consisting entirely of females and the median age of the enrolled patients was 54 (45–59.8) years. Written informed consent was obtained from the participants. This study was approved by the institutional review board of Hiroshima University (number: E-2019-9241) and conforms with the Declaration of Helsinki. The patient cohort was enrolled between August 2019 and September 2021. Lymphedema was diagnosed by histological examination and indocyanine green (ICG) lymphography (Figures S4A and S4B).^57^ The recruitment criteria were as follows: (a) more than 2 years passed since cancer treatment; (b) no active infection; (c) no tumor recurrence or metastasis; (d) no history of comorbid medical disorders (heart failure, renal failure, hepatic failure, endocrine abnormality, immunological disease); (e) unilateral lymphedema; and (f) no history of having undergone LVA. We excluded patients and HCs with a history of COVID-19 from this study based on the questionnaire due to the possible influence on immune function. Data on the type of cancer that caused lymphedema, duration of edema, the radiation therapy used for cancer, frequency of cellulitis, the Campisi clinical staging of lymphedema,^16^ ICG dermal backflow stage,^17^ and number of LVA were obtained for each patient. The EL index was calculated by dividing the sum of the squares of the circumference of five areas of the affected extremity by the BMI. The severity of the EL index was calculated by dividing the difference in the EL index affected and contralateral extremity by the affected extremity EL value for each case, as follows: [affected EL index - contralateral EL index]/(affected EL index) × 100. The rate of improvement of the EL index was calculated by dividing the difference in the EL index before and after surgery by the preoperative EL value for each case, as follows: [preoperative EL index - postoperative EL index]/(preoperative EL index) × 100. Peripheral blood samples were collected from 21 patients with lymphedema and 20 HCs. For post-LVA samples, peripheral blood samples were collected 12 months after LVA.

### Method details

#### LVA operative technique

A small amount (0.25 mg/0.1 mL) of ICG (Diagnogreen Injection, Daiichi Pharmaceutical, Tokyo, Japan) was injected subcutaneously into the first web space in upper extremity lymphedema, lateral malleolus, and the lateral side of the superior edge of the knee in lower extremity lymphedema. Furthermore, 12–18 h after the injection, we observed the ICG lymphography results using a near-infrared imaging device (Photodynamic Eye; Hamamatsu Photonics, Hamamatsu, Japan) and classified them into types Ⅰ to Ⅴ, as reported previously.^17^ LVA was performed under local anesthesia in all cases along a linear pattern or along the ulnar side of the upper extremity in upper extremity lymphedema, and greater saphenous vein course in lower extremity lymphedema in the area of the dermal backflow pattern.^58^ The LVA procedures were performed in an end-to-end manner using 11-0 or 12-0 nylon micro sutures under a surgical microscope (Figures S4C and S4D).^9^

#### Preparation of peripheral blood mononuclear cells (PBMCs)

A 15-mL aliquot of fresh peripheral blood was collected from each patient and HCs. PBMCs were isolated with Lymphoprep gradient (Axis-Shiel PoC AS, Oslo, Norway) and used for each experiment after confirming viability >95%, as determined by trypan blue staining.

#### Flow cytometry analysis

Cell surface marker staining of freshly isolated PBMCs was performed using appropriate combinations of fluorescein-conjugated anti-human antibodies. Briefly, cell suspensions (1 × 10^6^ to 2 × 10^6^) were incubated with a cocktail of the antibodies in the dark for 30 min at 4°C. Intracellular staining of Foxp3 was performed with the FOXP3 Fix/Perm Buffer Set (Biolegend) according to the manufacturer’s instructions. After surface labeling, PBMCs were permeabilized in 1 mL of fixation/permeabilization buffer at 20°C for 45 min in the dark. The samples were then stained with the anti-human FoxP3 monoclonal antibody and incubated in the dark for 30 min at 20°C. For analysis of cytokine production, PBMCs were stimulated with PMA (50 ng/mL) and ionomycin (750 ng/mL) in the presence of brefeldin A (10 μg/mL) (BioLegend). Cells were surface-stained, fixed, permeabilized, and stained with anti-cytokine antibodies. Isotype-matched control antibodies were used to establish the background levels of staining. 7-Aminoactinomycin D and Zombie-NIR (Biolegend) were used to identify and exclude dead T cells. Fluorescence-activated cell sorting (FACS) analysis was performed using a FACS Canto II flow cytometer (Becton Dickinson and Company (BD), San Jose, CA, USA). Data were analyzed using DIVA software (BD) and FlowJo (FlowJo LLC, San Jose, CA, USA).

Gating strategy and expression profiles of PD-1, Tim-3, and Lag-3 in peripheral CD4^+^ and CD8^+^ T cells are described in Figure S5A. It is now accepted that Treg cells are heterogeneous in phenotype and function, with three distinct subpopulations identified in the human peripheral blood^34^: Treg I (CD45RA^+^ FOXP3lo), Treg II (CD45RA^-^ FOXP3hi), and Treg III (CD45RA^-^ FOXP3lo) cells (Figure S5B). The surface expression of T-cell memory markers, including CCR7, CD45RO, CD95, CD49d, and CXCR3, were used to judge the differentiation status of cells. We examined the compartmentalization of naïve cells (CCR7^+^CD45RO^-^CD95^-^CXCR3^-^), memory T cells with a naïve phenotype (Tmnp; CCR7^+^CD45RO^-^CD95^-^CD49d^+^CXCR3^+^), stem cell-like memory T cells (Tscm; CCR7^+^CD45RO^-^CD95^+^), central memory cells (CCR7^+^CD45RO^+^), effector memory cells (CCR7^-^CD45RO^+^), and terminal effectors (CCR7^-^CD45RO^-^). The gating strategy for differentiated memory T cells is described in Figure S5C. Cytokine production in T cells is described in Figure S5D. The rate of change in a specific marker of T cells was calculated similarly to the rate of improvement in the EL index.

#### RNA extraction

Total RNA was isolated from PBMCs and purified with RNeasy Plus Universal Mini Kit (Qiagen, Hilden, Germany) according to the manufacturer’s instructions. RNA quantity and purity were measured with Agilent 2200 TapeStation (Agilent Technologies, Palo Alto, CA).

#### Unbiased amplification of TCR genes and sequencing

Next-generation sequencing analysis was performed with an unbiased TCR repertoire analysis technology developed by Repertoire Genesis Inc. (Osaka, Japan). In brief, unbiased adaptor-ligation PCR was performed according to the previous report.^55^^,^^56^ Total RNA was converted to complementary DNA (cDNA) with Superscript III reverse transcriptase (Invitrogen, Carlsbad, CA). BSL-18E primer containing polyT_18_ and a *Not*I site was used for cDNA synthesis. After cDNA synthesis, double-strand (ds)-cDNA was synthesized with *E. coli* DNA polymerase I (Invitrogen), *E. coli* DNA Ligase (Invitrogen), and RNase H (Invitrogen). ds-cDNAs were blunted with T4 DNA polymerase (Invitrogen). P10EA/P20EA adaptor was ligated to the 5ʹ end of the ds-cDNA and then cut with *Not*I restriction enzyme. After removal of the adaptor and primer with MinElute Reaction Cleanup kit (Qiagen), PCR was performed with KAPA HiFi DNA Polymerase (Kapa Biosystems, Woburn, MA) using constant region-specific 1st PCR and P20EA primers. PCR conditions were as follows: 98°C (20 s), 65°C (30 s), and 72°C (1 min) for 20 cycles. The second PCR was performed with 2nd PCR and P20EA primers using the same PCR conditions. Amplicons were prepared by amplification of the second PCR products using Tag PCR and P22EA-ST1-R primers. After PCR amplification, index (barcode) sequences were added by amplification with Nextera XT index kit v2 setA or setD (Illumina, San Diego, CA). The indexed amplicon products were mixed in an equal molar concentration and quantified by a Qubit 3.0 Fluorometer (Thermo Fisher Scientific, Waltham, MA). Sequencing was performed using the Illumina Miseq paired-end platform (2 × 300 bp).

#### TCR repertoire analysis

All the paired-end reads were classified by index sequences. Assignment of sequences was performed by determining sequences with the highest identity in a data set of reference sequences from the international ImMunoGeneTics information system® (IMGT) database. Data processing, assignment, and data aggregation were automatically performed using repertoire analysis software Repertoire Genesis (RG), which was originally developed by Repertoire Genesis Inc. (Osaka, Japan). RG is a program for sequence homology searches using BLASTn, an automatic aggregation program, a graphics program for gene usage, and CDR3 length distribution. Sequence identities at the nucleotide level between query and entry sequences were automatically calculated. Parameters that increased sensitivity and accuracy [E-value threshold, minimum kernel, high-scoring segment pair (HSP) score] were carefully optimized for respective repertoire analysis. Nucleotide sequences of CDR3 regions ranging from conserved cysteine at position 104 (Cys104) of IMGT nomenclature to conserved phenylalanine or tryptophan at position 118 (Phe118 or Trp118) were translated to deduce amino acid sequences. A unique sequence read (USR) was defined as a sequence read having no identity in assignment of gene segments and deduced amino acid sequence of CDR3 with the other sequence reads. The copy number of identical USR were automatically counted using RG software in each sample and then ranked in order of the copy number. Percentage occurrence frequencies of sequence reads with V and J genes in total sequence reads were calculated.

### Quantification and statistical analyses

Data are shown as median (IQR). JMP statistical software (SAS Institute Inc., Cary, NC, USA) was used for all statistical analyses. Non-parametric, independent, and paired continuous variables were compared using Mann–Whitney U test and Wilcoxon’s rank-sum test, respectively. The Chi-square test was used for categorical variables. Statistical analyses between lymphedema and post-LVA were primarily conducted, and subsidiary analyses were conducted between lymphedema and HCs, and post-LVA and HCs. Spearman’s rank correlation was used to evaluate the association between the quantitative indicators. Two-sided p-values <0.05 were considered statistically significant.

## Data Availability

The raw data of TCR β sequences has been deposited ad Zenodo (Zenodo: https://doi.org/10.5281/zenodo.7854980), and is publicly available as of the date of publication. This paper does not report original code. Any additional information required to reanalyze the data reported in this paper is available from the lead contact upon request.
