# Supplementary material for: Peripheral T cell profiling reveals downregulated exhaustion marker and increased diversity in lymphedema post-lymphatic venous anastomosis
Source: iScience. 2023 May 6;26(6):106822. doi: 10.1016/j.isci.2023.106822 (PMC10212982; doi:10.1016/j.isci.2023.106822)
Supplement: Document S1. Figures S1–S5 and Tables S1–S9 [file mmc1.pdf]

**iScience, Volume *26***

## Supplemental information

# Peripheral T cell profiling reveals downregulated exhaustion marker and increased diversity in lymphedema post-lymphatic venous anastomosis

**Hirofumi Imai, Takakazu Kawase, Shuhei Yoshida, Toshiro Mese, Solji Roh, Asuka Fujita, Toshio Uchiki, Ayano Sasaki, Shogo Nagamatsu, Atsushi Takazawa, Tatsuo Ichinohe, and Isao Koshima**

**Supplemental information**

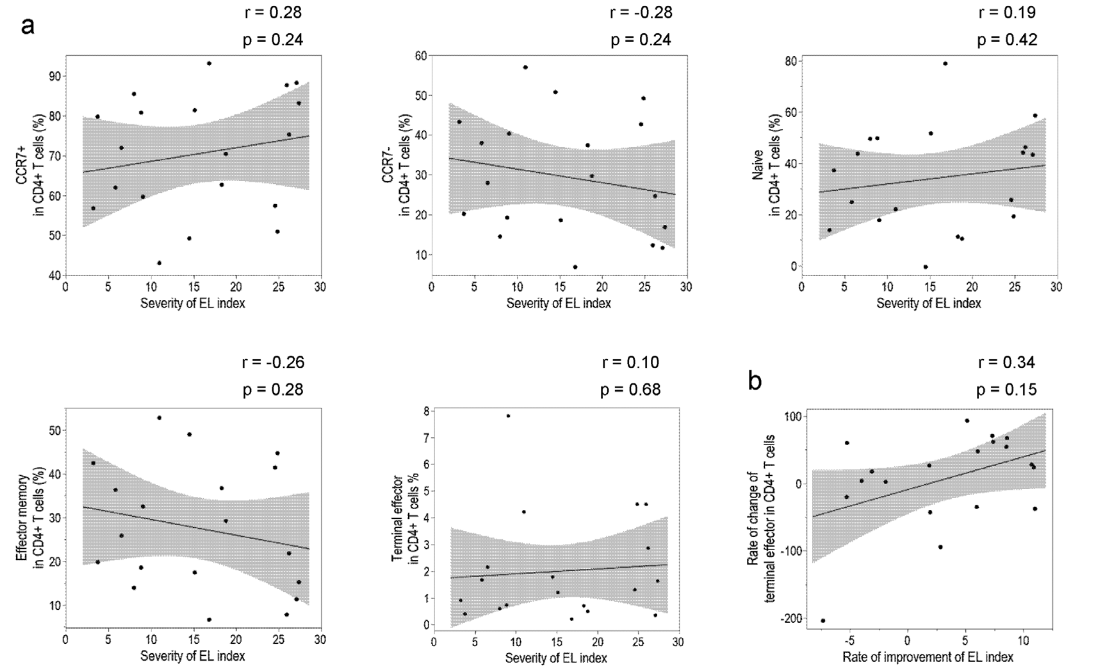


Figure S1. Correlation between Naïve memory phenotype in CD4$^+$ T cells and clinical features; Related to Figure 5. (**a**) Correlation between Naïve memory phenotype in CD4$^+$ T cells and severity of extremity lymphedema (EL) index. (**b**) Correlation between rate of change of terminal effector (TE) and rate of improvement of EL index.

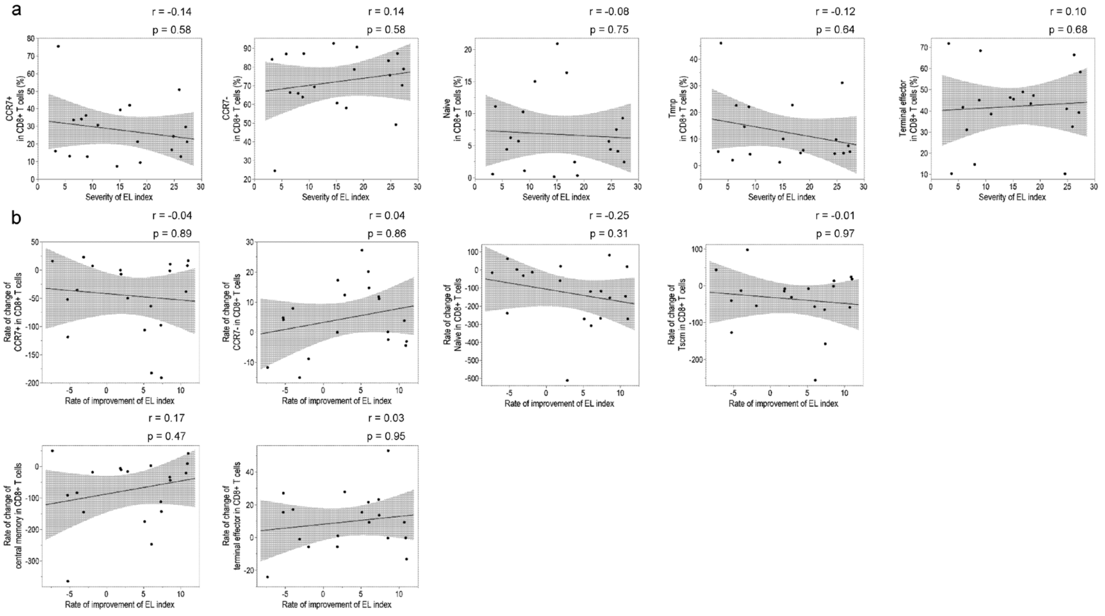


Figure S2. Correlation between naïve memory phenotype in CD8$^+$ T cells and clinical features; Related to Figure 6. (**a**) Correlation between naïve memory phenotype in CD8$^+$ T cells and severity of extremity lymphedema (EL) index. (**b**) Correlation between rate of change of Naïve memory phenotype and rate of improvement of EL index.

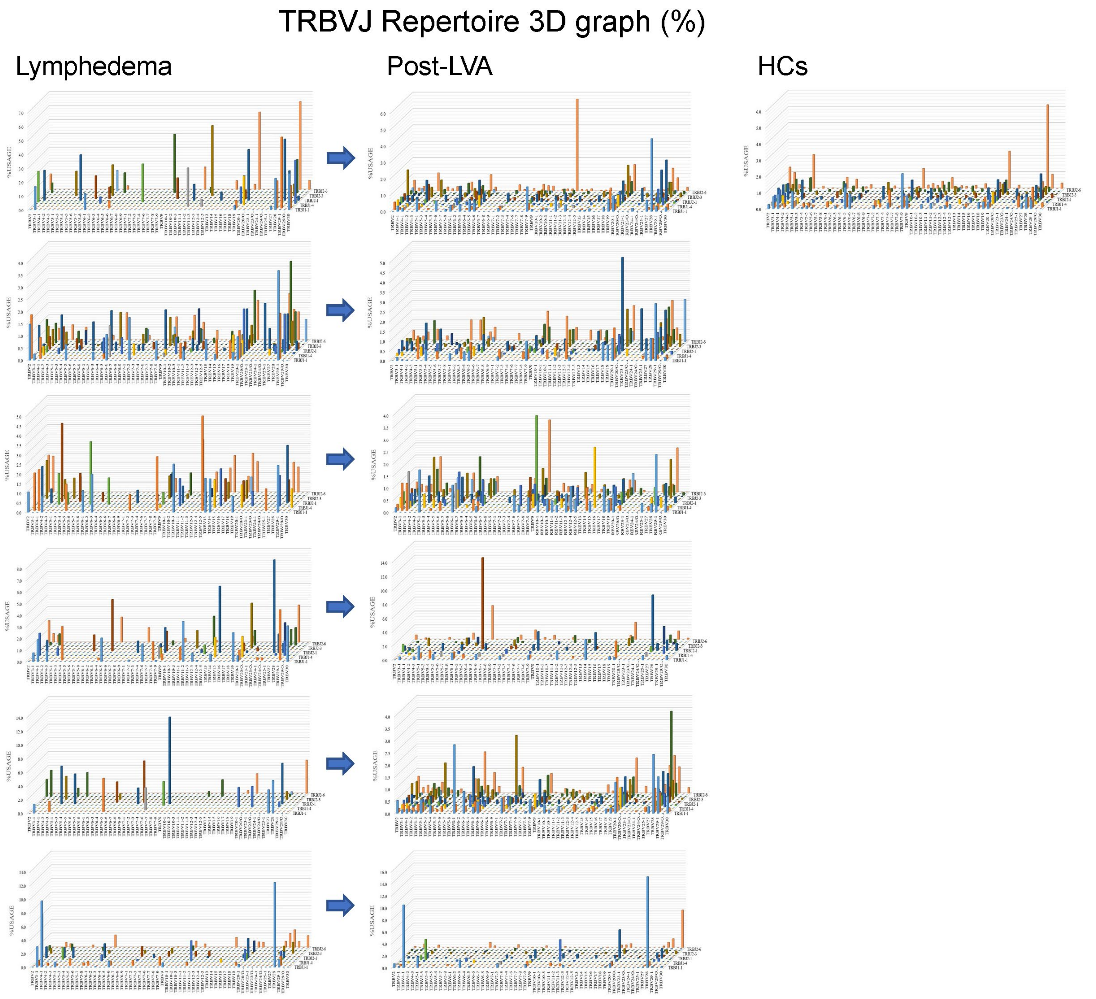


Figure S3. Variety of TRBV and J combination in lymphedema, post-lymphatic venous anastomosis (LVA), and healthy control (HC) samples; Related to Figure 9.

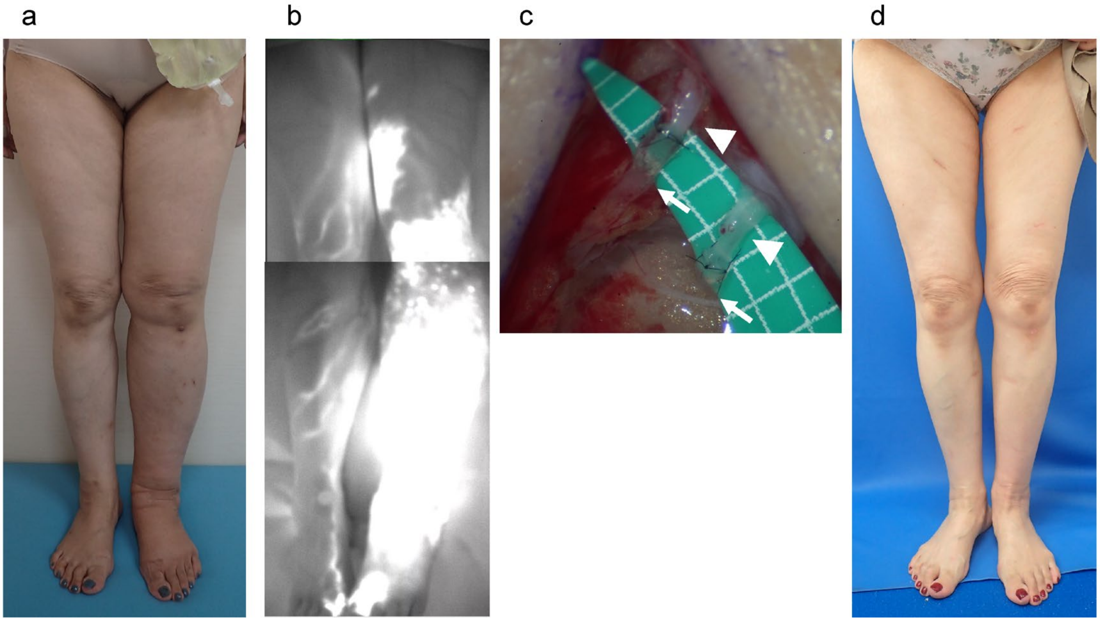


Figure S4. Edematous condition of patients with left lower limb lymphedema; Related to Figure 1. (**a**). Compared with those on the right side, the circumferences are higher on the left side. (**b**). The indocyanine green lymphology shows marked dermal backflow on the left side, while a linear pattern can be noted on the right side. (**c**) Lymphatic venous anastomosis (LVA) was performed for the patient. Arrow indicates the lymphatic channel, and the arrowhead indicates the vein. The scale of the background sheet is 1 mm. (**d**) The circumference in the left lower extremity was decreased at 1-year post-LVA.

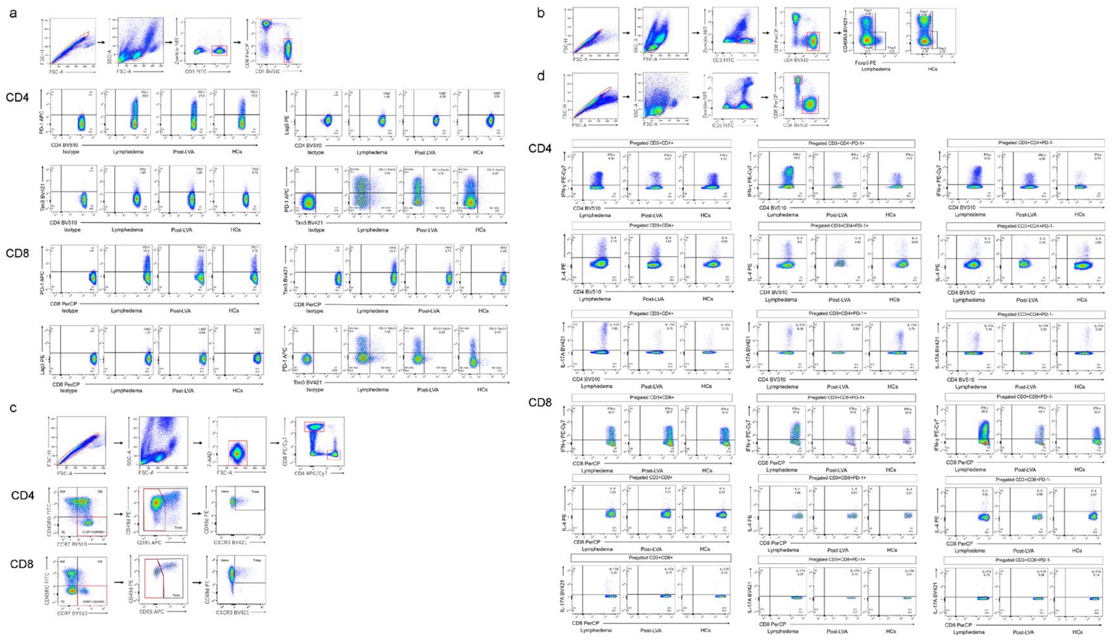


Figure S5. Gating strategy and expression profiles of T cells; Related to Figures 2–8. (**a**) Exhaustion marker, (**b**) Treg, (**c**) Naïve and memory phenotype, and (**d**) cytokine production are demonstrated.

Table S1. Clinical characteristics of patients; Related to Figure 1.

| Demographic | Lymphedema (n = 21) | Healthy controls (n = 20) | p Value |
|---|---|---|---|
| Sex, n (%) | | | |
| Male | 0 (0) | 0 (0) | 1.0 |
| Female | 21 (100) | 20 (100) | |
| Age, y, median (IQR) | 54 (45–59.8) | 51 (44.5–63) | 0.55 |
| BMI, kg/m², median (IQR) | 20.7 (19.2–25.1) | N/A | N/A |
| Upper or lower extremity lymphedema, n (%) | | N/A | N/A |
| Upper extremity lymphedema | 4 (19.0) | | |
| Lower extremity lymphedema | 17 (81.0) | | |
| Etiology, n (%) | | None | None |
| Uterine cancer | 14 (66.7) | | |
| Ovarian cancer | 2 (9.5) | | |
| Bladder cancer | 1 (4.8) | | |
| Breast cancer | 4 (19.0) | | |
| Duration of edema, y, median (IQR) | 2.7 (0.5–7.5) | None | None |
| Chemotherapy, yes, n (%) | 16 (76.2) | None | None |
| Radiotherapy, yes, n (%) | 7 (33.3) | None | None |
| Cellulitis episode, yes, n (%) | 7 (33.3) | None | None |
| ISL stage, n (%) | | N/A | N/A |
| Stage 1 | 0 (0) | | |
| Stage 2a | 5 (23.8) | | |
| Stage 2b | 13 (61.9) | | |
| Stage 3 | 3 (14.3) | | |
| Dermal backflow stage, n (%) | | N/A | N/A |
| Stage I | 0 | | |
| Stage II | 4 (19.0) | | |
| Stage III | 7 (33.3) | | |
| Stage IV | 4 (19.0) | | |
| Stage V | 4 (19.0) | | |

BMI: Body mass index. IQR: Interquartile range. ISL: International Society of Lymphology

Table S2. Comparison of extremity lymphedema index and leukocyte characteristics between lymphedema, post-lymphatic venous anastomosis (LVA), and healthy controls (HCs); Related to Figure 1.

| Demographic, median (IQR) | Lymphedema (n = 21) | Post-LVA (n = 21) | HCs (n = 20) | p-value | | |
|---|---|---|---|---|---|---|
| | | | | Lymphedema vs. Post-LVA | HCs vs. Lymphedema | Post-LVA vs. HCs |
| UEL index | 126.7 (106.0–159.8) | 125.4 (108.9–143.2) | N/A | 0.25 | N/A | N/A |
| LEL index | 265.5 (245.2–307.7) | 251.7 (241.9–263.5) | N/A | 0.01 | N/A | N/A |
| Severity of EL index | 16.8 (8.4–25.4) | 12.3 (4.1–18.1) | N/A | < 0.01 | N/A | N/A |
| Leukocyte count, n/μl | 5300 (4535–6045) | 4515 (3705–5298) | 5110 (3885–5920) | 0.27 | 0.48 | 0.57 |
| Lymphocyte count, n/μl | 1790 (1132–2010) | 1410 (1184.5 – 1939) | 1810 (1551–2229) | 0.50 | 0.44 | 0.14 |
| CD4/8 ration in $CD3^+$ T cells | 1.5 (0.9–2.5) | 1.5 (0.9–2.0) | 1.8 (1.4–2.5) | 0.07 | 0.21 | 0.08 |

IQR: Interquartile range. UEL: Upper extremity lymphedema index. LEL: Lower extremity lymphedema index

Table S3. Comparison of PD-1, Tim-3, Lag-3, and PD-1$^{+}$Tim3$^{+}$ expression (%) on CD4$^{+}$ and CD8$^{+}$ T cells between lymphedema, post-lymphatic venous anastomosis (LVA), and healthy controls (HCs); Related to Figures 2 and 3.

| Demographic %, median (IQR) | Lymphedema (n = 21) | Post-LVA (n = 21) | HCs (n = 20) | p-value | | |
|---|---|---|---|---|---|---|
| | | | | Lymphedema vs. Post-LVA | HCs vs. Lymphedema | Post-LVA vs. HCs |
| PD-1$^{+}$ in CD3$^{+}$4$^{+}$ T cells | 30.7 (19.8–39.1) | 27.1 (17.6–35.4) | 19.9 (13.9–24.7) | 0.03 | < 0.01 | 0.02 |
| Tim-3$^{+}$ in CD3$^{+}$4$^{+}$ T cells | 1.4 (0.9–3.0) | 1.0 (0.7–1.4) | 1.3 (0.6–1.5) | < 0.01 | 0.29 | 0.51 |
| Lag-3$^{+}$ in CD3$^{+}$4$^{+}$ T cells | 0.6 (0.4–0.8) | 0.4 (0.3–0.7) | 0.4 (0.3–0.6) | 0.06 | 0.01 | 0.40 |
| PD-1$^{+}$Tim-3$^{+}$ in CD3$^{+}$4$^{+}$ T cells | 0.7 (0.4–1.1) | 0.3 (0.2–0.6) | 0.3 (0.2–0.6) | < 0.01 | 0.04 | 0.69 |
| PD-1$^{+}$ in CD3$^{+}$8$^{+}$ T cells | 17.1 (11.7–25) | 15.9 (10.2–19.4) | 17.5 (9.4–20.6) | 0.01 | 0.26 | 0.66 |
| Tim-3$^{+}$ in CD3$^{+}$8$^{+}$ T cells | 3.5 (1.8–6.5) | 2.2 (1.3–4.0) | 3.4 (2.5–4.2) | 0.01 | 0.57 | 0.09 |
| Lag-3$^{+}$ in CD3$^{+}$8$^{+}$ T cells | 1.2 (0.7–1.7) | 0.7 (0.5–1.6) | 0.8 (0.5–1.3) | 0.15 | 0.09 | 0.75 |
| PD-1$^{+}$Tim-3$^{+}$ in CD3$^{+}$8$^{+}$ T cells | 0.6 (0.3–1.0) | 0.2 (0.1–0.5) | 0.3 (0.2–0.7) | 0.01 | 0.08 | 0.56 |

IQR: Interquartile range

Table S4. Comparison of percentage of Treg subsets among $CD3^{+}CD4^{+}$ T cells (%) between lymphedema, post-lymphatic venous anastomosis (LVA), and healthy controls (HCs); Related to Figure 4.

| Demographic %, median (IQR) | Lymphedema (n = 21) | Post-LVA (n = 21) | HCs (n = 20) | p-value | | |
|---|---|---|---|---|---|---|
| | | | | Lymphedema vs. Post-LVA | HCs vs. Lymphedema | Post-LVA vs. HCs |
| Treg in $CD3^{+}4^{+}$ T cells | 5.3 (3.0–7.4) | 5.1 (2.9–6.1) | 2.5 (1.4–4.6) | 0.17 | <0.01 | 0.03 |
| Treg Ⅰ in $CD3^{+}4^{+}$ T cells | 0.6 (0.4–0.9) | 0.5 (0.3–0.7) | 0.4 (0.3–1.1) | 0.09 | 0.26 | 0.67 |
| Treg Ⅱ in $CD3^{+}4^{+}$ T cells | 1.1 (0.8–2.1) | 1.4 (0.9–2.6) | 0.5 (0.3–0.9) | 0.25 | < 0.001 | < 0.0001 |
| Treg Ⅲ in $CD3^{+}4^{+}$ T cells | 2.8 (1.8–4.5) | 2.5 (1.7–3.3) | 1.6 (0.9–3.0) | 0.06 | 0.02 | 0.14 |

IQR: Interquartile range

Table S5. Comparison of naïve and memory phenotype frequencies in $CD4^+$ and $CD8^+$ T cells between lymphedema, post-lymphatic venous anastomosis (LVA), and healthy controls (HCs); Related to Figures 5, 6.

| Demographic %, median (IQR) | Lymphedema (n = 21) | Post-LVA (n = 21) | HCs (n = 20) | p-value | | |
|---|---|---|---|---|---|---|
| | | | | Lymphedema vs. Post-LVA | HCs vs. Lymphedema | Post-LVA vs. HCs |
| $CCR7^+$ in $CD4^+$ T cells | 72.0 (57.4–83.2) | 74.1 (57.8–79.5) | 83.6 (76.5–87.2) | 0.31 | 0.02 | < 0.01 |
| $CCR7^-$ in $CD4^+$ T cells | 28.0 (16.8–42.7) | 26 (20.1–42.2) | 16.4 (12.9–23.5) | 0.32 | 0.02 | < 0.01 |
| Naive in $CD4^+$ T cells | 37.2 (17.8–49.5) | 35.1 (17.6–40.0) | 51.1 (39.6–57.0) | 0.05 | 0.01 | < 0.01 |
| Tscm in $CD4^+$ T cells | 3.1 (2.1–4.8) | 4.2 (2.7–5.4) | 2.8 (2.1–3.9) | 0.06 | 0.51 | 0.03 |
| Tmnp in $CD4^+$ T cells | 3.4 (1.5–6.2) | 8.1 (2.7–13.6) | 3.1 (1.5–9.1) | 0.12 | 0.78 | 0.17 |
| Central memory in $CD4^+$ T cells | 27.8 (20.0–32.0) | 29.3 (22.1–31.8) | 24.8 (18.2–29.1) | 0.25 | 0.31 | 0.19 |
| Effector memory in $CD4^+$ T cells | 25.8 (15.2–41.4) | 24.9 (18.5–38.2) | 16.0 (12.2–21.8) | 0.46 | 0.02 | < 0.01 |
| Terminal effector in $CD4^+$ T cells | 1.3 (0.6–2.9) | 2.4 (0.9–4.5) | 0.7 (0.4–1.3) | 0.02 | 0.17 | 0.01 |
| $CCR7^+$ in $CD8^+$ T cells | 24.3 (13.1–36.1) | 18.6 (9.2–36.5) | 41.5 (35.9–54.6) | 0.02 | < 0.001 | < 0.001 |
| $CCR7^-$ in $CD8^+$ T cells | 75.7 (63.9–87) | 81.4 (63.6–90.8) | 58.6 (45.4–64.2) | 0.02 | < 0.001 | < 0.001 |
| Naive in $CD8^+$ T cells | 5.7 (2.4–10.3) | 2.2 (0.7–7.6) | 10.8 (6.5–23.1) | < 0.01 | < 0.01 | < 0.001 |
| Tscm in $CD8^+$ T cells | 3.4 (2.1–5.5) | 3.0 (2.2–3.4) | 3.5 (2.0–4.6) | 0.04 | 1.0 | 0.16 |
| Tmnp in $CD8^+$ T cells | 6.6 (4.7–22.2) | 7.9 (3.3–23.0) | 22.3 (11.5–29.3) | 0.42 | < 0.01 | 0.01 |
| Central memory in $CD8^+$ T cells | 3.6 (2.4–6.3) | 2.6 (1.8–3.6) | 4.1 (3.0–5.2) | < 0.01 | 0.61 | 0.01 |
| Effector memory in $CD8^+$ T cells | 30.9 (16.7–43.5) | 26.1 (19.2–33.3) | 31.2 (19.4–35.6) | 0.22 | 0.95 | 0.82 |
| Terminal effector in $CD8^+$ T cells | 43.4 (32.5–48.9) | 49.3 (30.9–58.6) | 23.5 (17.3–34.1) | 0.02 | < 0.01 | < 0.01 |

IQR: Interquartile range

Tscm: stem cell-like memory T cell

Tmnp: memory T cells with naïve phenotype

Table S6. Comparison of cytokine production in $CD4^+$ and $CD8^+$ T cells between lymphedema, post-lymphatic venous anastomosis (LVA), and healthy controls (HCs); Related to Figures 7 and 8.

| Demographic %, median (IQR) | Lymphedema (n = 10) | Post-LVA (n = 10) | HCs (n = 10) | p-value | | |
|---|---|---|---|---|---|---|
| | | | | Lymphedema vs. Post-LVA | HCs vs. Lymphedema | Post-LVA vs. HCs |
| IFN-γ in $CD3^+4^+$ T cells | 10.8 (7.1–20.3) | 9.7 (6.1–20.1) | 6.9 (4.6–9.7) | 0.18 | 0.03 | 0.28 |
| IFN-γ in $CD3^+4^+PD\text{-}1^+$ T cells | 30.1 (19.7–40.9) | 24.7 (19.0–26.1) | 20.6 (16.0–25.1) | 0.04 | 0.03 | 0.44 |
| IFN-γ in $CD3^+4^+PD\text{-}1^-$ T cells | 5.8 (3.6–11.1) | 5.9 (2.8–12.5) | 3.7 (3.0–5.0) | 0.29 | 0.05 | 0.38 |
| IL-4 in $CD3^+4^+$ T cells | 2.1 (1.5–5.8) | 2.3 (2.2–3.8) | 1.9 (1.2–2.3) | 0.47 | 0.28 | 0.12 |
| IL-4 in $CD3^+4^+PD\text{-}1^+$ T cells | 4.9 (4.3–11.1) | 4.5 (3.0–5.5) | 4.5 (3.5–5.5) | 0.34 | 0.25 | 0.92 |
| IL-4 in $CD3^+4^+PD\text{-}1^-$ T cells | 1.4 (0.8–3.9) | 1.8 (1.3–2.9) | 1.1 (0.8–1.5) | 0.29 | 0.37 | 0.10 |
| IL-17A in $CD3^+4^+$ T cells | 2.7 (1.8–3.3) | 1.6 (0.9–2.3) | 1.7 (1.5–2.2) | 0.01 | 0.06 | 0.60 |
| IL-17A in $CD3^+4^+PD\text{-}1^+$ T cells | 5.5 (3.6–6.3) | 4.0 (2.7–4.5) | 6.2 (4.9–6.8) | 0.04 | 0.29 | < 0.01 |
| IL-17A in $CD3^+4^+PD\text{-}1^-$ T cells | 1.3 (0.8–2.5) | 0.8 (0.4–1.6) | 0.7 (0.5–1.1) | 0.01 | 0.10 | 0.79 |
| IFN-γ in $CD3^+8^+$ T cells | 47.1 (38.1–56.6) | 41.2 (40.3–49.8) | 31.3 (19.5–44.8) | 0.23 | 0.02 | 0.05 |
| IFN-γ in $CD3^+8^+PD\text{-}1^+$ T cells | 66.2 (59.5–70.5) | 52.6 (43.0–61.1) | 64.6 (42.5–75.3) | 0.07 | 0.76 | 0.32 |
| IFN-γ in $CD3^+8^+PD\text{-}1^-$ T cells | 45.9 (34.5–54.8) | 41.0 (39.0–47.9) | 27.6 (17.4–40.3) | 0.27 | 0.01 | 0.02 |
| IL-4 in $CD3^+8^+$ T cells | 1.8 (0.8–2.6) | 1.6 (0.5–2.4) | 1.0 (0.8–1.4) | 0.34 | 0.16 | 0.38 |
| IL-4 in $CD3^+8^+PD\text{-}1^+$ T cells | 2.3 (0.9–2.7) | 2.0 (1.3–2.2) | 2.1 (1.2–2.7) | 0.34 | 0.87 | 0.44 |
| IL-4 in $CD3^+8^+PD\text{-}1^-$ T cells | 1.7 (0.7–2.6) | 1.5 (0.5–2.4) | 1.0 (0.7–1.2) | 0.40 | 0.20 | 0.24 |
| IL-17A in $CD3^+8^+$ T cells | 0.5 (0.2–0.6) | 0.2 (0.1–0.4) | 0.2 (0.1–0.5) | 0.16 | 0.33 | 0.66 |
| IL-17A in $CD3^+8^+PD\text{-}1^+$ T cells | 0.7 (0.5–1.2) | 0.4 (0.2–0.8) | 0.7 (0.3–0.9) | 0.10 | 0.50 | 0.37 |
| IL-17A in $CD3^+8^+PD\text{-}1^-$ T cells | 0.4 (0.1–0.6) | 0.1 (0.1–0.4) | 0.2 (0.1–0.4) | 0.16 | 0.29 | 0.79 |

IQR: Interquartile range

Table S7. Comparison of diversity index of T cell receptor between lymphedema, post-lymphatic venous anastomosis (LVA), and healthy controls (HCs); Related to Figure 9.

| Demographic, median (IQR) | Lymphedema (n = 6) | Post-LVA (n = 6) | HCs (n = 5) | P value | | |
|---|---|---|---|---|---|---|
| | | | | Lymphedema vs. Post-LVA | HCs vs. Lymphedema | Post-LVA vs. HCs |
| Total read, n | 223228 (156631–333851) | 235896 (154459–379691) | 336850 (249651–431229) | 0.22 | 0.12 | 0.24 |
| Functional read, n | 122939 (75361–184743) | 135626 (75706–193075) | 185662 (134533–226968) | 0.28 | 0.17 | 0.32 |
| In flame read, n | 118995 (70013–181235) | 130535 (72726–188194) | 175301 (127220–214288) | 0.16 | 0.24 | 0.41 |
| Unique read, n | 6596 (5515–9933) | 8532 (7497–10447) | 11358 (10681–20184) | 0.28 | 0.03 | 0.04 |
| Shannon Weaver index | 4.7 (4.3–5.4) | 6.1 (5.4–6.9) | 6.1 (5.8–6.6) | 0.02 | 0.01 | 0.86 |
| Inverse Simpson's index | 44.1 (30.6–95.1) | 200.5 (47.2–287) | 151.0 (132.0–299.5) | 0.02 | 0.03 | 1.00 |
| Pielou's evenness | 0.56 (0.50–0.61) | 0.67 (0.60–0.75) | 0.67 (0.63–0.69) | 0.02 | < 0.01 | 1.00 |
| Diversity Evenness 50 | 0.4 (0.2–0.6) | 1.2 (0.4–2.7) | 1.1 (0.7–1.2) | 0.03 | 0.01 | 0.72 |

IQR: Interquartile range

Table S8. Top 50 increased TCR β clones in post-lymphatic venous anastomosis compared with that in lymphedema; Related to Figure 10.

| TRBV | TRBJ | CDR3 | Amount reads of change |
|---|---|---|---|
| 9 | 2-1 | CASSVGQFNEQFF | 14897 |
| 7-3 | 2-2 | CASIREYTGELFF | 7204 |
| 28 | 2-1 | CASSIQGNEQFF | 7012 |
| 5-1 | 2-1 | CASSLEPGGPQFF | 6254 |
| 6-5 | 1-2 | CASNYKGGNYGYTF | 5768 |
| 30 | 2-7 | CAWSVTDNEQYF | 5723 |
| 12-4 | 2-3 | CASSLGWADTQYF | 5342 |
| 5-5 | 2-1 | CASSPGDSHNEQFF | 5182 |
| 30 | 1-1 | CAWFADGQNTEAFF | 5157 |
| 20-1 | 2-7 | CSAREEVGGDEQYF | 4957 |
| 5-4 | 1-2 | CASSEWDSGSIYGYTF | 4891 |
| 20-1 | 2-3 | CSALRGSSGGRDTQYF | 4748 |
| 28 | 1-1 | CASSSLVGFMNTEAFF | 4660 |
| 14 | 2-1 | CASSPDRARQFF | 4623 |
| 7-9 | 2-5 | CASSLQGETQYF | 4453 |
| 3-1 | 2-5 | CASSRRLATQETQYF | 4365 |
| 7-9 | 1-6 | CASNGGSHSPLHF | 4129 |
| 5-6 | 2-5 | CASSAVTTQETQYF | 4059 |
| 27 | 1-1 | CASRRTGEPNTEAFF | 4013 |
| 5-1 | 2-3 | CASSFQGLSTDTQYF | 3954 |
| 7-6 | 1-3 | CASSLGTANSGNTIYF | 3918 |
| 29-1 | 2-7 | CSAIASGSWEQYF | 3885 |
| 29-1 | 1-1 | CSVVTGSTEAFF | 3661 |
| 23-1 | 1-5 | CATGGSQGNQPQHF | 3589 |
| 6-6 | 2-2 | CASTQTATGELFF | 3575 |
| 11-2 | 1-1 | CASSLDRGKNTEAFF | 3508 |
| 12-4 | 2-5 | CASSLQARREETQYF | 3448 |
| 12-5 | 1-2 | CASGHGPGTGYYGYTF | 3432 |
| 6-5 | 1-1 | CASSYSIPGAGTEAFF | 3399 |
| 19 | 1-5 | CASSMTAVGGNQPQHF | 3335 |
| 20-1 | 2-7 | CSAFGGGLRSYEQYF | 3296 |

| | | | |
|---|---|---|---|
| 20-1 | 2-1 | CSAGSLSYNEQFF | 3163 |
| 4-1 | 1-1 | CASSYDRLLDTEAFF | 3147 |
| 2 | 2-7 | CASRGTVYEQYF | 3111 |
| 29-1 | 2-7 | CSVAVHTTYEQYF | 3084 |
| 5-6 | 2-1 | CASSPPGGGPHEQFF | 3065 |
| 20-1 | 1-4 | CSARASGGPNEKLFF | 3004 |
| 2 | 2-5 | CASSEERETQYF | 2968 |
| 7-3 | 1-6 | CASSLNAGANSPLHF | 2935 |
| 11-2 | 1-3 | CASSQGSMGFGTIYF | 2916 |
| 14 | 1-4 | CASSQRGTGINEKLFF | 2874 |
| 7-9 | 2-2 | CASAPGTLTGELFF | 2859 |
| 7-2 | 2-1 | CASSLYGGGGRNEQFF | 2814 |
| 13 | 2-5 | CASSPWTSGVQETQYF | 2800 |
| 30 | 1-1 | CAWVPGPSSTEAFF | 2767 |
| 7-8 | 1-2 | CASSRTGIYYGYTF | 2713 |
| 7-2 | 1-1 | CASSLRTGEAFF | 2711 |
| 2 | 1-1 | CASTSWTTGDAFF | 2669 |
| 29-1 | 1-1 | CSSRWGAEAFF | 2615 |
| 29-1 | 1-2 | CSVEAMDRGRNYGYTF | 2613 |

Table S9. Top 50 reduced TCR β clones in post-lymphatic venous anastomosis compared with that in lymphedema; Related to Figure 10.

| TRBV | TRBJ | CDR3 | Amount reads of change |
|---|---|---|---|
| 6-5 | 2-2 | CASSYSGNTGELFF | 17473 |
| 11-2 | 2-7 | CASSLTDQGYEQYF | 6265 |
| 6-5 | 2-7 | CASSSDGFYEQYF | 5097 |
| 28 | 1-1 | CASMDRGNTEAFF | 4438 |
| 14 | 1-1 | CASSQDRIEAFF | 4289 |
| 7-9 | 1-6 | CASSSHESGGVSSPLHF | 3324 |
| 28 | 1-1 | CASSPPDRGAFF | 3178 |
| 20-1 | 2-1 | CSARDPPPSGRGNEQFF | 2973 |
| 30 | 2-7 | CAWSPLGLADLRDNEQYF | 2601 |
| 7-6 | 2-7 | CASSPGYEQYF | 2545 |
| 5-1 | 1-1 | CASSLAQGKHTEAFF | 2458 |
| 11-2 | 1-1 | CASSDSDKLNTEAFF | 2385 |
| 7-9 | 2-1 | CASSLIGVSSYNEQFF | 2327 |
| 20-1 | 1-2 | CSARGASRAYGYTF | 2171 |
| 5-4 | 1-2 | CASSRKLTGYTF | 2095 |
| 2 | 1-6 | CASRTGQSYNSPLHF | 2063 |
| 6-1 | 2-3 | CASSVDHFTDTQYF | 2043 |
| 30 | 2-6 | CAGQGVLGANVLTF | 2039 |
| 4-3 | 2-3 | CASSQDLGGRSTDTQYF | 2012 |
| 19 | 1-6 | CASSQDRNSPLHF | 1961 |
| 29-1 | 1-5 | CSVAGVNQPQHF | 1904 |
| 6-3 | 2-4 | CASSYSSSGGTSVAKNIQYF | 1851 |
| 6-1 | 1-1 | CASSEKENTEAFF | 1828 |
| 29-1 | 1-1 | CSVGTGNTEAFF | 1827 |
| 5-5 | 2-1 | CASSLGWSSGRVSYNEQFF | 1810 |
| 6-6 | 2-1 | CASRNGGPNNEQFF | 1795 |
| 20-1 | 1-2 | CSARGDVTDSGDLFNYGYTF | 1782 |
| 6-1 | 1-1 | CASSVVGLNTEAFF | 1730 |
| 6-1 | 1-2 | CASSDGRELLKLYGYTF | 1688 |
| 6-1 | 2-4 | CASSVRQEAGKNIQYF | 1619 |
| 29-1 | 2-7 | CSVASDIHSYEQYF | 1595 |

| | | | |
|---|---|---|---|
| 30 | 2-1 | CACNELGNKGAEQFF | 1584 |
| 20-1 | 2-5 | CSAGPGLAGQETQYF | 1569 |
| 20-1 | 2-1 | CSAPRAGIADNEQFF | 1536 |
| 6-1 | 2-1 | CASSDGEQGGKFF | 1534 |
| 7-9 | 2-1 | CASSPTGGARYNEQFF | 1508 |
| 20-1 | 2-7 | CSAIQGANEQYF | 1503 |
| 28 | 2-5 | CASWRLRQETQYF | 1502 |
| 7-2 | 2-3 | CASSFLPDSGMSTDTQYF | 1501 |
| 6-6 | 1-1 | CATDRIQWKTNTEAFF | 1498 |
| 25-1 | 1-6 | CASSESVGDSPLHF | 1481 |
| 6-1 | 2-7 | CATGTLFSYEQYF | 1478 |
| 25-1 | 2-1 | CASSDRRQRSSYNEQFF | 1478 |
| 5-1 | 1-2 | CASSLEAAGGSGYTF | 1460 |
| 5-6 | 1-3 | CASSLGQAGGNTIYF | 1432 |
| 4-1 | 1-1 | CASSQEEQGIKNTEAFF | 1408 |
| 7-2 | 2-5 | CASSRTGTSGGLQETQYF | 1402 |
| 5-1 | 1-6 | CASRTGGYADSPLHF | 1391 |
| 20-1 | 1-2 | CSARAQDYGYTF | 1371 |
| 5-5 | 1-1 | CASSWGGEFAEAFF | 1366 |
